# Privacy-preserving pangenome graphs

**DOI:** 10.1038/s41467-026-77591-0

**Published:** 2026-09-14

**Authors:** Jacob Blindenbach, Shaunak Soni, Gamze Gürsoy

**Affiliations:** 1 Department of Computer Science, Columbia University, New York, USA; 2 Department of Biomedical Informatics, Columbia University, New York, USA; 3 New York Genome Center, New York, USA; 4 Wayne Hills High School, New Jersey, USA; 5 Department of Applied Mathematics and Theoretical Physics, University of Cambridge, Cambridge, UK

**Keywords:** Computational biology and bioinformatics, Genomics

## Abstract

The human pangenome reference, often represented as a graph, promises to capture genetic diversity across populations, but open release of individual haplotypes raises significant privacy concerns, including risks of re-identification and inference of sensitive traits. To address these challenges, we introduce PanMixer, a framework for privacy-preserving pangenome graph releases that selectively obfuscates an individual’s haplotypes while retaining the utility of the reference graph. PanMixer formulates the privacy-utility trade-off as a knapsack problem, where privacy risk is quantified using information-theoretic measures and utility is measured using graph properties. Using the recently released draft human pangenome graphs, we show that PanMixer robustly reduces re-identification risk under linkage attacks and genome reconstruction attempts. We also show that PanMixer preserves the accuracy of key downstream applications, including allele frequency estimation, linkage disequilibrium analysis, and read mapping. By addressing privacy concerns, PanMixer enables the inclusion of individuals, particularly those from underrepresented populations, who might otherwise be reluctant to contribute but seek representation in future genomic studies. Our results provide both a practical tool and a generalizable framework for balancing privacy and utility in future large-scale pangenome references.

## Abstract (web summary)

Here the authors present PanMixer, a framework that obfuscates haplotype paths in pangenome graphs to reduce privacy risk while preserving downstream utility.

## Introduction

The human pangenome represents a comprehensive reference framework that integrates genomic sequences from diverse populations to capture the full spectrum of human genetic variation^1–3^. Unlike the current linear reference genome, which is derived predominantly from a limited number of individuals of European ancestry^4^, the pangenome incorporates structural variants, insertions, deletions, and population-specific haplotypes that were previously underrepresented or absent^1^. This expanded representation improves read mapping^5^, variant calling^6^, and functional annotation^7^ across ancestries, and consequently enhances the accuracy of genomic analyses. By providing a more inclusive and complete model of human genetic diversity, the pangenome has the potential to advance studies of evolution, disease susceptibility, and precision medicine, and ultimately facilitate equitable applications of genomics in global health^1–3^.

Pangenome graphs are the graphical representation of pangenome references by compressing multiple genomes into nodes of shared DNA sequences connected by edges that capture haplotype-specific paths^1^. In 2023, the first human pangenome was released^1^, along with graph representations generated with two state-of-the-art tools: Pangenome Graph Builder (PGGB)^8^ and Minigraph-Cactus^9^. The Human Pangenome Reference Consortium (HPRC) is now expanding this resource to many more individuals^10^, a scale designed to further reduce reference bias and enhance studies of human evolution, disease, and ancestry^11^. Public availability of the human pangenome reference is critical for accelerating scientific discovery and democratizing data access.

The open release of new individual haplotype information may raise public concern without some form of privacy protections in place to minimize the risk of re-identifying sample donors. Furthermore, individuals may not be willing to participate in the human pangenome reference construction efforts under a fully open access model, but these individuals may be critical to include in a pangenome reference resource that is intended to represent all possible haploblocks in the human genome. Such concerns are not merely hypothetical, but are grounded in historical cases where inadequate attention to privacy and consent led to lasting harm. For example, Henrietta Lacks’s cells (HeLa), which were taken and shared without consent, contributed to biomedical breakthroughs such as the polio vaccine^12^ and advances in cancer treatment^13,14^, but also created lasting mistrust among African Americans^15,16^. Similarly, misuse of Native American DNA such as the unauthorized use of Havasupai Tribe samples for research beyond diabetes led to lawsuits, damaged trust, and reduced willingness to participate in genetics studies^17–19^. To ensure the pangenome represents all populations, especially those historically underrepresented, privacy must be front of mind and built in from the start.

**Fig. 1:**
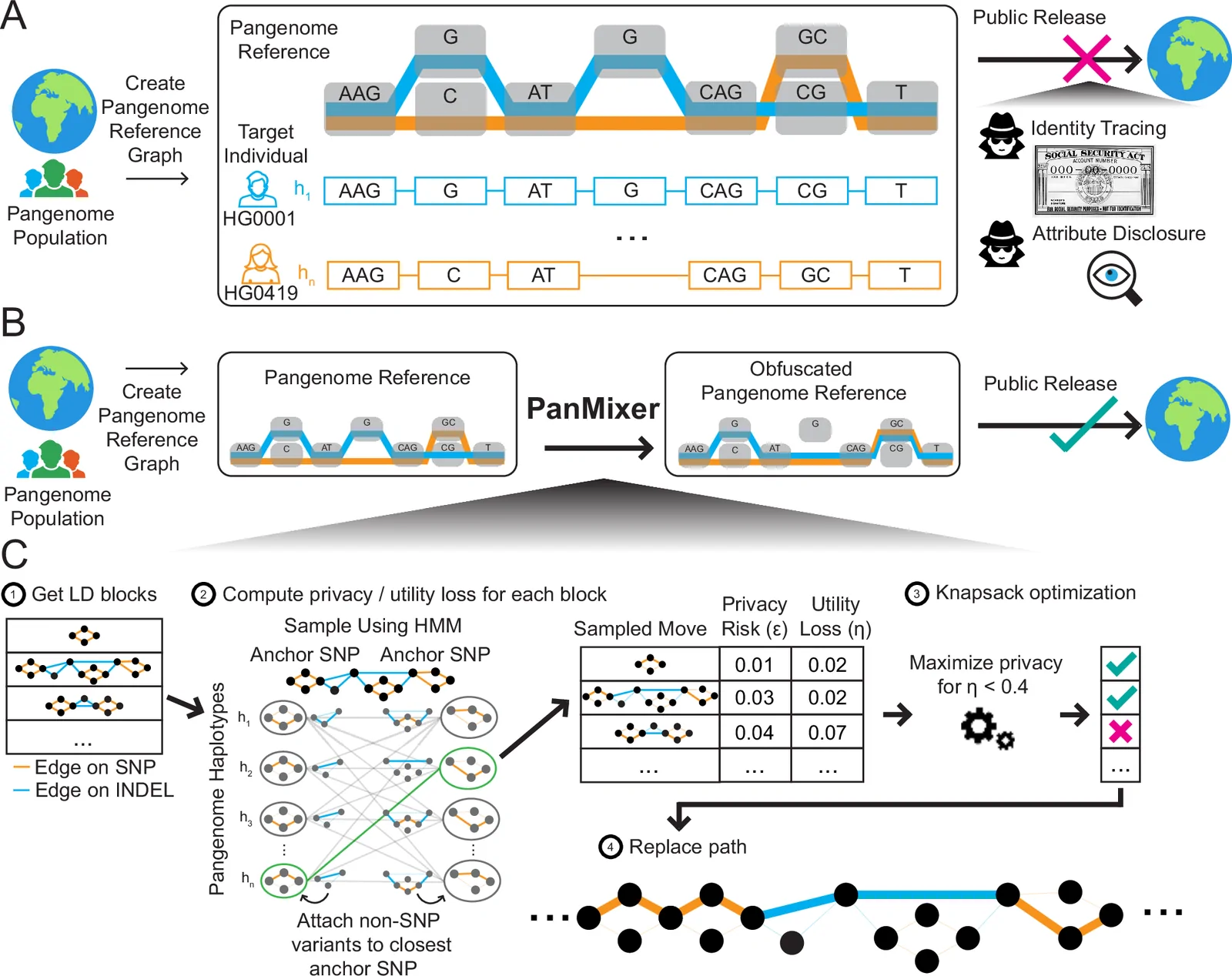
Privacy-preserving pangenome release by haplotype path obfuscation. **A** In the current pangenome release model, a cohort of patients is gathered, sequenced, and combined into a pangenome reference using a pangenome graph builder. This pangenome graph, where individual haplotypes are represented as paths traversing a sequence of nodes containing varying lengths of DNA, is released to the public. Although releasing both the graph and the haplotype paths enables comprehensive representation of population-level variation, an individual’s haplotype paths may reveal private or rare variants that risk re-identification or sensitive attribute inference. **B** PanMixer updates this release model by obfuscating the target individual’s paths before release to improve the privacy of these target individuals. **C** PanMixer (1) identifies LD blocks, (2) computes privacy and utility metrics for potential obfuscation moves sampled via a Li-Stephens-like HMM to preserve LD structure, (3) selects an optimal subset of moves via knapsack optimization given a utility budget, and (4) applies these moves to produce the released obfuscated paths.

In the current pangenome release model, an individual’s genome is represented as haplotype paths through the pangenome graph. Following an individual’s haplotype path through the graph fully reconstructs their genome sequence (Fig. 1A). While this enables scientific utility, it also creates privacy risks: rare or unique variants along a haplotype path can serve as identifiers, enabling re-identification or linkage to sensitive phenotypic traits, as previous studies have shown^20^. Thus, a central challenge emerges: how can individuals contribute to a public pangenome reference in a way that preserves both representation and protects against privacy risks?

To address these privacy concerns, we introduce PanMixer, an obfuscation tool that enables participation in the pangenome reference while mitigating re-identification risk. PanMixer selectively obfuscates haplotype paths of target individuals to preserve privacy while maintaining the structure and the analytical utility of the graph. We formalized the privacy-utility trade-off as a knapsack problem and, using an integer programming approach, identified the optimal set of paths that minimizes privacy risk while maximizing graph utility for downstream analyses (Fig. 1B). Privacy is quantified using pointwise mutual information, while utility is measured by a path-based edit distance. We further show that our information-theoretic privacy metric correlates with the success of linkage attacks and our utility metric correlates with the performance of multiple downstream applications, including allele frequency (AF) analysis, linkage disequilibrium (LD) analysis, and read mapping.

## Results

### PanMixer is a framework designed to balance genomic privacy and data utility in pangenome graphs

Safeguarding individual privacy while maintaining the utility of genomic resources is a central challenge for the next generation of pangenome projects. PanMixer addresses this challenge by introducing a principled framework for selectively obfuscating haplotype paths within a pangenome graph. The input to PanMixer is the haplotype paths of a target individual whose privacy we want to protect, and the output is a privacy-preserving version of the pangenome graph. PanMixer achieves this by defining a set of obfuscation moves on a target individual’s haplotype paths. These moves modify the nodes and edges that the haplotype path of a certain individual traverses inside the graph. Each move improves the privacy of the target individual at a utility loss to the pangenome graph. At its core, PanMixer solves an optimization problem, finding a subset of obfuscation moves that achieves the desired level of privacy for the target individual while minimizing the loss of utility of the pangenome graph. We formulate this optimization as the 0–1 knapsack problem. In the 0–1 knapsack problem, one seeks to choose a subset of items, each with a weight and value, that maximizes the total value without exceeding the knapsack’s capacity^21^. Analogously, each “item” is an obfuscation move, the “weight” corresponds to the utility loss this move causes to the pangenome graph, and the “value” represents the privacy risk to the target individual (Fig. 1C). In PanMixer, we measure utility loss using the Weighted Path Edit Distance (WPED) between the original and obfuscated paths, and privacy risk using the Pointwise Mutual Information (PMI) between them. We define these terms formally in the following sections.

Under the hood, each obfuscation move is defined on an LD block. These blocks are computed from SNPs on the linear reference backbone of the graph (what we will refer to as top-level SNPs)^22^. The obfuscation outcome of each block is obtained from either population-level allele frequencies or through a Hidden Markov Model (HMM) from the Li-Stephens model^23^ if there are enough top-level SNPs to model the haplotypes in the LD block. Once we determine all the possible moves, we use OR-Tools^24^, a 0–1 knapsack linear optimization solver, to find the best set of moves to take that achieves a certain level of privacy for the target individual while minimizing the utility loss of the pangenome graph. These moves replace the target individual’s haplotype paths in the original pangenome graph with paths that prevent private information leakage (Fig. 1C).

### Quantifying privacy and utility

We quantify privacy risk (denoted as *ϵ*) using PMI between the original haplotype path (*h*) and the obfuscated haplotype path (h^*^):

1 $$\,{\rm{PMI}}\,(h,{h}^{*})\equiv \log \frac{p(h,{h}^{*})}{p(h)p({h}^{*})},$$

where *p*(*h*) and *p*(h^*^) denote the probabilities of observing the original and obfuscated haplotype paths in the population, respectively, and *p*(*h*, h^*^) denotes their joint probability. We define the joint probability *p*(*h*,h^*^) blockwise over independent LD blocks (*b*_1_, ⋯ , *b*_*k*_). For blocks that are not obfuscated, the released and original haplotypes are identical, implying the joint probability of that block equals the marginal probability ($p({h}_{{b}_{j}},{h}_{{b}_{j}}^{*})=p({h}_{{b}_{j}})$). Conversely, for obfuscated blocks, the released haplotype is sampled independently of the original, yielding a factorizable joint distribution ($p({h}_{{b}_{j}},{h}_{{b}_{j}}^{*})=p({h}_{{b}_{j}})p({h}_{{b}_{j}}^{*})$). This construction leads directly to the blockwise decomposition of PMI derived in the Supplementary Lemma 1. A comprehensive list of all mathematical notations used in this paper is provided in Supplementary Table S1.

High PMI corresponds to high privacy risk as the obfuscated path is strongly statistically dependent on the original path. This makes it easier for an attacker observing the obfuscated path to reconstruct the original path. By contrast, a small PMI or even a PMI(*h*, h^*^) = 0 means that the obfuscated path is nearly or is statistically independent of the original and conveys little to nothing about the original path.

For utility (denoted by *η*), we use the Weighted Path Edit Distance (WPED), defined as the minimum cost of all edits required to transform *h* into h^*^. Formally:

2 $$\,{\rm{WPED}}\,(h,{h}^{*})\equiv {\mathop{\min }\limits_{{\delta }_{1},\cdots \,,{\delta }_{k}\in {{\mathcal{M}}}}}\, \sum _{j=1}^{k}c({\delta }_{j}),$$

where ${{\mathcal{M}}}$ defines the set of all possible obfuscation moves, *δ*_*j*_ is an obfuscation move, and *c*(*δ*_*j*_) is its cost.

WPED is conceptually similar to the Weighted Graph Edit Distance (WGED), but instead of measuring edits between nodes and edges of two graphs, it measures edits between the paths in the original graph and the graph with the obfuscated genomes. A low WPED indicates few edits (high utility), whereas a high WPED indicates many edits (low utility).

#### Privacy and utility loss in practice

From our equations above, computing privacy risk requires estimating haplotype path probabilities *p*(*h*). Since no large-scale dataset of path frequencies exists, we cannot compute these directly. Instead, we represent haplotypes by the sequence of variants they traverse. In this representation, the graph is decomposed into top-level variants and nested variants (variants occurring within the span of larger variants, such as a SNP inside a large insertion). Each haplotype path corresponds to a sequence of alleles across these variants.

This variant-centric view allows us to approximate path probabilities from population allele frequencies. We use the 1000 Genomes dataset for top-level SNP frequencies, and the PanGenie callset^6^ for structural and nested variants, derived from sequencing reads of all 1000 Genomes individuals aligned to the draft pangenome graph^1^. These AF estimates are then integrated with an HMM that accounts for LD between variants to get an overall probability for each haplotype path.

Privacy risk is therefore tightly linked to allele frequencies, with rare variants contributing disproportionately to the privacy risk and being obfuscated first. This dependency ensures that PanMixer behaves conservatively for individuals from underrepresented populations or for novel variants where external reference data may be sparse. When external frequency estimates are unavailable, PanMixer derives estimates from the graph itself. Thus, an individual with an LD block that contains a novel variant will have a high privacy risk for that LD block, and its corresponding obfuscation move will be prioritized for selection.

For utility, we again rely on the variant-centric representation of the graph. Obfuscation moves are restricted to allele modifications within LD blocks (including nested variants). Each move has cost $c({\delta }_{j})={\sum }_{v\in \Delta }\frac{1}{\,{\rm{support}}\,(v)}$, where Δ is the set of variants altered by the move, and support(*v*) is the number of haplotypes traversing variant *v*. This cost function closely approximates AF changes, and for LD blocks with multiple or nested variants, the total utility loss is roughly proportional to the number of edits. Consider a single SNP (Supplementary Fig. S1) on a linear reference backbone such as GRCh38, where an individual’s haplotype follows the reference path. Redirecting it through the alternate allele changes its AF by ${L}_{1}({{\rm{AF}}}_{v},{{\rm{AF}}}_{{v}^{*}})=\frac{1}{{\rm{support}}\,(v)},$ where *L*_1_ is the L1 loss function. The resulting utility loss is constant and does not depend on AF, in contrast to privacy risk, which scales with AF.

Because utility loss scales predictably with the number of edits, whereas privacy risk depends strongly on population allele frequencies, balancing the two requires a formal optimization framework. Each obfuscation move *δ*_*j*_ on an LD block *b*_*j*_ is associated with a utility loss *η*_*j*_ = *c*(*δ*_*j*_) and a privacy risk ${\epsilon }_{j}=-\log p({h}_{{b}_{j}})$ (derived in the Supplementary Lemma 2), where $p({h}_{{b}_{j}})$ is the population probability of haplotype block ${h}_{{b}_{j}}$. The total privacy risk of the full haplotype is then ${\epsilon }_{{{\rm{tot}}}}={\sum }_{j=1}^{k}{\epsilon }_{j}.$ Obfuscating block *b*_*j*_ removes its contribution *ϵ*_*j*_ from the total risk. We therefore define the *removed privacy risk* as $\rho ({{\bf{x}}})=\mathop{\sum }_{j=1}^{k}{\epsilon }_{j}{x}_{j},$ where **x** ∈ {0, 1}^*k*^ is a binary decision vector indicating which of the *k* possible obfuscation moves are applied. The realized privacy risk is *ϵ*_res_(**x**) = *ϵ*_tot_ − *ρ*(**x**). Minimizing the realized privacy risk is equivalent to maximizing the removed privacy risk. This yields the 0–1 knapsack formulation:

3 $${\mathop{{{\rm{maximize}}}}_{{{\bf{x}}}\in {\{0,1\}}^{k}}}\,\,\rho ({{\bf{x}}})=\sum _{j=1}^{k}{\epsilon }_{j}{x}_{j}\,\,\,{\rm{subject \, to}}\,\,\,\sum _{j=1}^{k}{\eta }_{j}{x}_{j}\le \Delta U$$

where Δ*U* is the maximum allowable utility loss. The objective is to maximize privacy by removing as much risk as possible, while ensuring that the total utility loss remains within budget.

### Symmetric privacy-utility tradeoffs reveal robustness of PanMixer optimization

**Fig. 2:**
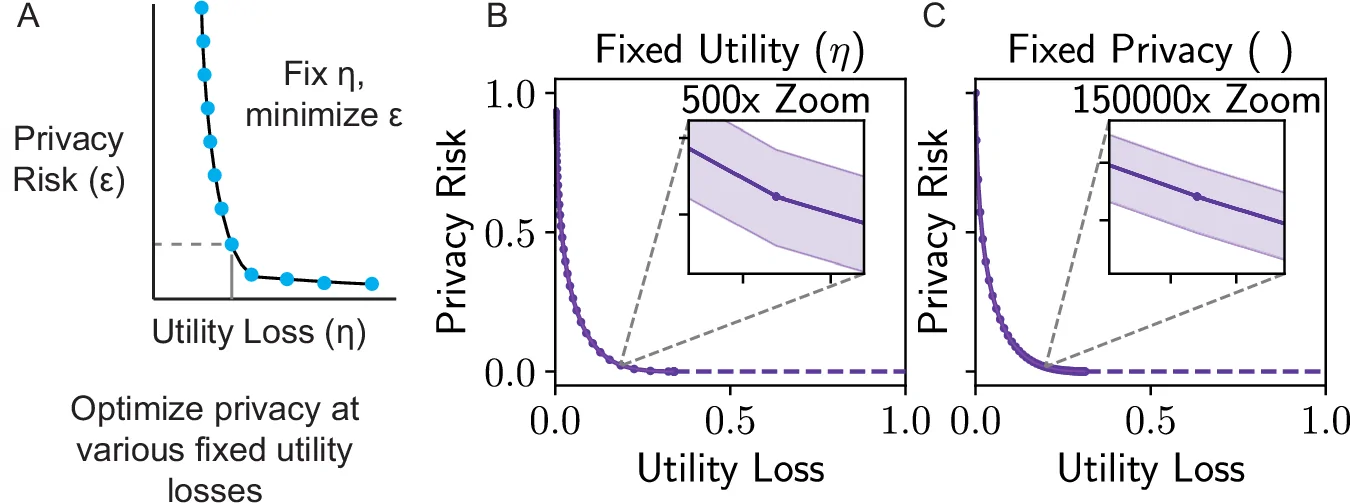
Privacy-utility tradeoff across individuals and ancestries. **A** Schematic of the privacy-utility tradeoff curve generation process. We picked various utility-loss capacities, and then minimized the privacy risk (*ϵ*) for a target individual constrained by that utility loss (*η*). **B** Privacy risk (*ϵ*) achieved by PanMixer across a range of fixed utility-loss levels, averaged over all individuals (*n* = 44). The solid line shows the mean across individuals; the shaded band denotes the full range (minimum to maximum) across individuals. The privacy risk has been normalized to [0, 1] by dividing by the total privacy risk, *ϵ*_tot_ (not having any obfuscation). **C** Reciprocal optimization: utility loss incurred under fixed privacy constraints. The solid line shows the mean across individuals (*n* = 44); the shaded band denotes the full range (minimum to maximum) across individuals. Tradeoff curves are nearly identical, confirming symmetry in the optimization problem and consistency across ancestries.

We generated and characterized the privacy-utility tradeoff in pangenome graph obfuscation by applying PanMixer 44 times for all 44 individuals in the HPRC v1 pangenome reference^1^ graph created using PGGB^8^. For each target individual, we defined a set of normalized utility loss levels ranging from 0 (unaltered reference) to 1 (maximally perturbed, where all variants are modified), and optimized for privacy (we have illustrated this process schematically in Fig. 2A). To ensure consistent comparisons across individuals, we similarly normalized the resulting privacy risk to the range [0, 1] by dividing by the total privacy risk of the individual’s original haplotypes (*ϵ*_tot_).

We observed a consistent concave tradeoff shape, with diminishing returns in privacy as utility loss increased (Fig. 2B). The apparent smoothness of the privacy-utility curves reflects the heterogeneous distribution of block-level privacy contributions rather than a trivial optimization, and no single operating point is privileged by design. The solid line denotes the mean privacy achieved across individuals, and the shaded region captures the full individual-level range. Stratifying by ancestry revealed almost no visible difference between the tradeoff curves (See Supplementary Fig. S2), indicating that our definitions of privacy and utility, as well as the optimization, generalize well across all populations.

We confirmed the robustness of the optimization by repeating the analysis under fixed privacy risk, optimizing instead for minimal utility loss (Fig. 2C). The resulting curves closely matched those from the privacy-optimized procedure, demonstrating the symmetry of the tradeoff and validating PanMixer’s consistency across optimizing for privacy and utility.

### Privacy metric aligns with linkage attack and genome reconstruction risk

Next, we show how our privacy metric is indicative of re-identification risk. To demonstrate this empirically, we evaluated the pangenome graphs produced by PanMixer under a linkage-based threat scenario.

First, we assume an adversary who has access to the released pangenome graph and possesses auxiliary information in the form of an external genotype database (e.g., the 1000 Genomes dataset). The adversary’s objective is re-identification: they aim to link a specific, obfuscated haplotype path within the graph to a known individual in their external database, thereby exposing their identity.

**Fig. 3:**
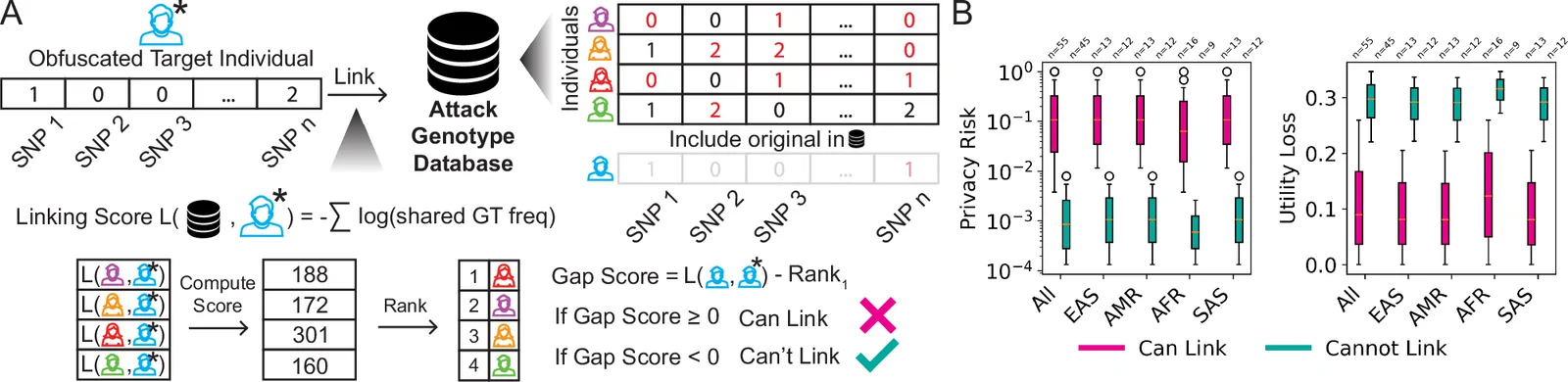
Validating privacy risk through re-identification attacks. **A** Schematic of a re-identification attack. An adversary attempts to link an obfuscated individual in the released pangenome graph to an entry in an external genotype database using a rarity-weighted matching score. **B** Box plots of privacy risk (*ϵ*) and utility loss (*η*) for individuals successfully linked versus not linked by the attacker. In both box plots, the center line is the median, box bounds are the 25th and 75th percentiles, whiskers extend to 1.5 × IQR, and points beyond are plotted as outliers; minima and maxima are the outermost plotted points. Higher privacy risk (*ϵ*) is associated with increased re-identification success, confirming that individuals who are successfully linked retain higher PMI values (less privacy) than those who are safe. This validates the empirical relevance of *ϵ* as a meaningful privacy metric.

As shown in Fig. 3A, the attacker attempts to link each obfuscated haplotype path to genomes in the 1000 Genomes Project Phase 3 callset^25^, using a rarity-weighted matching score that gives greater weight to the shared rare variants, similar to approaches used previously^26–29^. That is, the linkage score is defined as the negative sum of the log-frequencies of the shared genotypes, ensuring that shared rare variants contribute more to the linkage scores (see “Methods” for more details). We applied this attack to pangenome graphs obfuscated by PanMixer at varying privacy levels, ranging from 0 (perfect privacy) to 1 (no obfuscation). For each individual with genotypes in the callset, we extracted shared SNPs between the obfuscated path and the database entries, computed rarity scores, and ranked candidates in the database. Finally, we calculated a Gap Score, defined as the linking score between the original and obfuscated haplotypes minus the highest linking score found in the attack database; a re-identification was considered successful if the Gap Score was positive (>0) and unsuccessful if the score was negative (<0).

In this pangenome graph, linking attacks failed once the privacy risk was reduced below *ϵ *≤ *ϵ*_private_ = 0.002 (Fig. 3B). We define *ϵ*_private_ as the largest privacy risk value at which all obfuscated target individuals could no longer be linked. Importantly, *ϵ*_private_ is not universal as it depends on the specific graph, external genotype database, and target individuals. PanMixer provides a systematic way to compute *ϵ*_private_ for any given set of target individuals, pangenome graphs, and external genotype databases. In our experiments, reaching this privacy threshold corresponded to an average utility loss of 0.28 (on a scale where the maximum possible loss is 1).

Next, we evaluated a scenario analogous to a membership inference attack, in which an adversary possesses the full original genome of a target individual and seeks to determine whether that individual is represented in the pangenome. We modeled this attack by having the adversary attempt to link the target’s known genome to all haplotype paths in the pangenome graph using the same rarity-weighted matching metric explained in the re-identification attack. A linkage attack is considered successful when the score distribution contains a clear outlier: one candidate genome scores significantly higher than all others, making the attacker more likely to identify that candidate as the obfuscated genome and conclude that the individual is present in the pangenome. Conversely, obfuscation is successful when the true obfuscated genome does not emerge as the top-scoring outlier. In this case, either another candidate receives a higher linking score, or no candidate is clearly distinguishable, making the attacker more likely to misidentify the target or fail to identify them with confidence.

As shown in Supplementary Fig. S3, the linking score between each target’s original and obfuscated genome (red line) falls within the bulk of the distribution of linking scores against other subjects, indicating the obfuscated individual is indistinguishable from other candidates in the pangenome.

A different class of threats comes from genome reconstruction attacks, where an adversary tries to reconstruct obfuscated alleles from surrounding LD structure to either recover the original alleles or strengthen re-identification attacks. PanMixer obfuscates entire LD blocks rather than individual variants to minimize the ability of such an attacker. Genotype imputation methods^30–33^ rely on LD to infer missing alleles by exploiting correlations with surrounding variants. By splitting the haplotype paths into blocks with little to no LD between them, PanMixer makes the reconstruction of obfuscated LD blocks ineffective using traditional imputation techniques. We demonstrate this empirically by simulating an attacker attempting to reconstruct obfuscated haplotype paths using Beagle^30^ and found their accuracy is no better than simply guessing the major allele at each site, which is essentially equivalent to having no information about the true genotype (Supplementary Fig. S4). Thus, LD-based methods cannot recover additional information beyond what is already contained in the obfuscated block, simplifying both the obfuscation problem and our privacy analysis to work on a block-by-block basis.

To demonstrate the necessity of haplotype-level obfuscation, we compared PanMixer against a baseline strategy that only removes unique or private variants while leaving common variation intact^34^. As shown in Supplementary Fig. S5, this strategy failed to prevent re-identification, maintaining a positive Gap Score comparable to the original graph. This confirms that removing unique markers is insufficient for privacy; the unique combination of common alleles within a haplotype remains a quasi-identifier that PanMixer effectively disrupts.

### PanMixer obfuscation preserves utility in common pangenome downstream analysis

Utility, like privacy, must be measured in context. To this end, we assessed the impact of PanMixer’s obfuscations on three key downstream features of pangenome analysis: allele frequency spectra, linkage disequilibrium patterns, and read mapping quality.

#### Impact of obfuscation on AF spectra and linkage disequilibrium

We evaluated the fidelity of AF and LD in pangenome graphs with obfuscated paths produced by PanMixer. Specifically, we compared the change in AF and LD across all variants between the original pangenome graph and the pangenome graphs in which the genome of a target individual was obfuscated at varying levels of utility loss. We measure the fidelity of AF and LD in pangenome graphs using the following fidelity metric: Wasserstein divergence for the AF spectra difference and the L1 loss (sum of absolute differences) between LD matrices derived from the original and obfuscated graphs. LD was measured as pairwise *R*^2^ correlations between SNPs within sliding 5 kb windows.

**Fig. 4:**
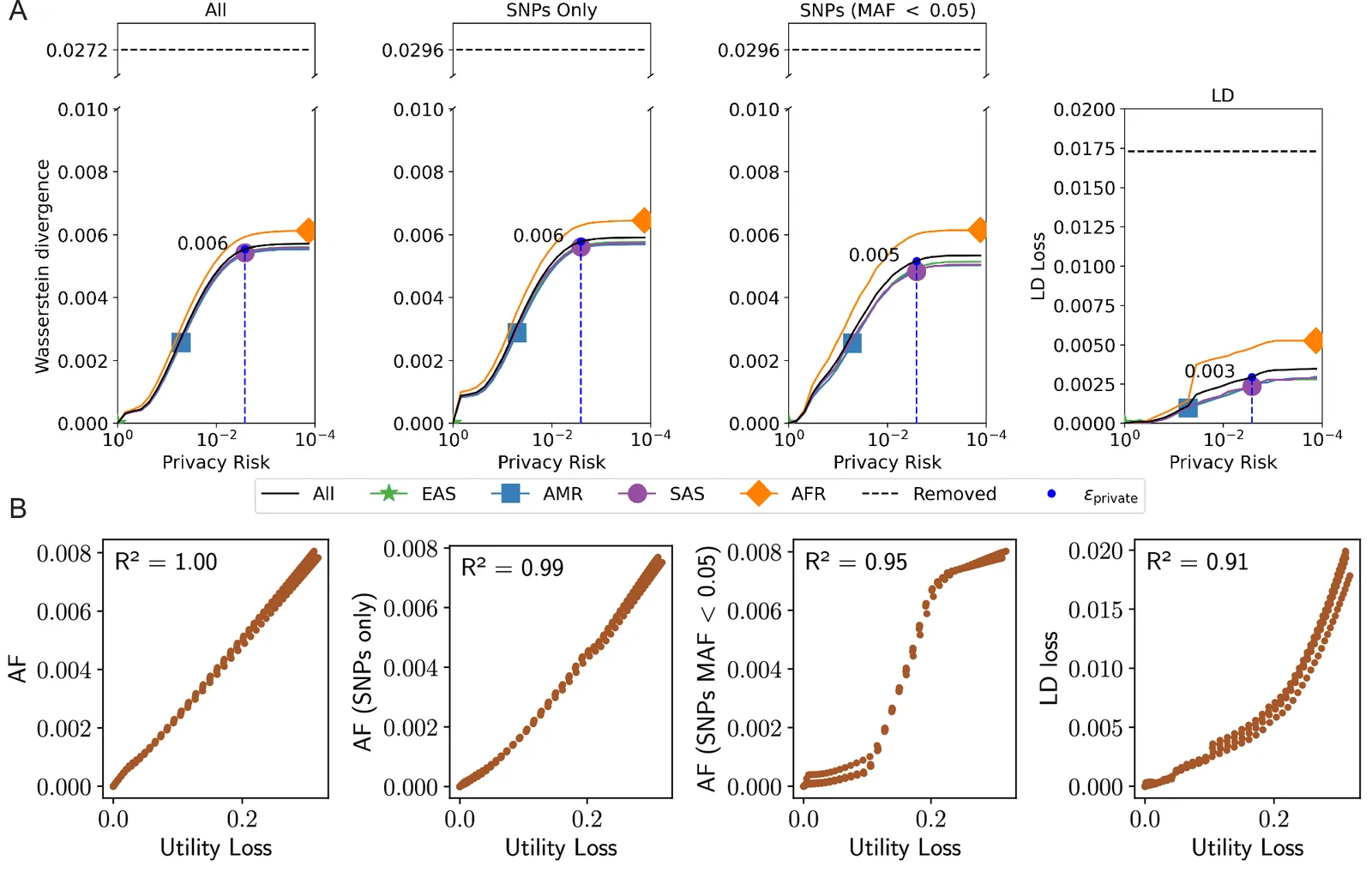
Downstream utility analysis for individuals within the pangenome reference. **A** Fidelity of allele frequency (AF; all, SNP, and rare SNP variants) and linkage disequilibrium (LD) from obfuscated pangenome graphs across privacy levels (*ϵ*). AF divergence (Wasserstein) remains below 0.008 across all *ϵ* but rises sharply under the removed baseline (complete removal of the target’s haplotypes). Likewise, the LD loss stays low (<0.006) for all targets (*ϵ*_private_). **B** Correlation between PanMixer’s utility-loss metric and empirical distortions in downstream statistics. Utility loss correlates strongly with both AF divergence (*R*^2^ = 1.00 across all variants; 0.99 for SNPs; 0.95 for rare SNPs) and LD decay (*R*^2^ = 0.91), supporting its validity as an indicator of downstream impact.

For AF spectra, we computed the Wasserstein divergence between the AF distributions of the original and edited graphs for all variants and variant types. Across various values of privacy risk, the divergence remained below 0.008 (Fig. 4A), indicating minimal impact on allele frequencies. To benchmark utility preservation, we compared PanMixer against a ’Removed’ baseline, where target individuals were entirely excluded from the graph. This baseline represents the maximum possible utility loss for a given individual. This baseline showed substantially higher divergence. For PanMixer edited graphs, at *ϵ*_private_ = 0.002 (the privacy risk threshold where linking attacks failed), the Wasserstein divergence was 0.006 for all alleles, 0.006 for SNPs only, and 0.005 for rare SNPs (population MAF < 0.05), which is close to 5x smaller than the divergence observed when target haplotypes were removed entirely, 0.027. For pairwise LD values, the LD matrix was on average 0.003 off at *ϵ*_private_, lower than the 0.017 observed for the complete genome removal baseline.

#### Utility loss is correlated with AF and LD divergence

Next, we show the usefulness of our utility metric by showing its correlation with the fidelity metrics (Fig 4B). Utility loss was strongly correlated with AF divergence, with an *R*^2^ of 1.00 for all variants, 0.99 for SNPs only, and 0.95 for rare SNPs (MAF < 0.05). The correlation with LD decay was also strong (*R*^2^ = 0.91).

#### Obfuscation preserves read mapping quality

We next assessed whether the inclusion of an obfuscated genome in the pangenome graph affects its utility for read mapping that originates from sequencing of an external genome. We aligned 1000 Genomes individuals^25^ not included in the pangenome graphs to both PanMixer-produced graphs (at *ϵ*_private_, which protects target patients against linking) and to the original graph as a baseline using Giraffe^5^ (v1.68.0). Notably, we aligned reads using three graph constructions commonly applied in practice to improve mapping performance: the full unfiltered graph, an allele-frequency-filtered graph (variants with alternative allele frequency < 10% removed), and a personalized graph with 8 haplotypes^35^.

**Fig. 5:**
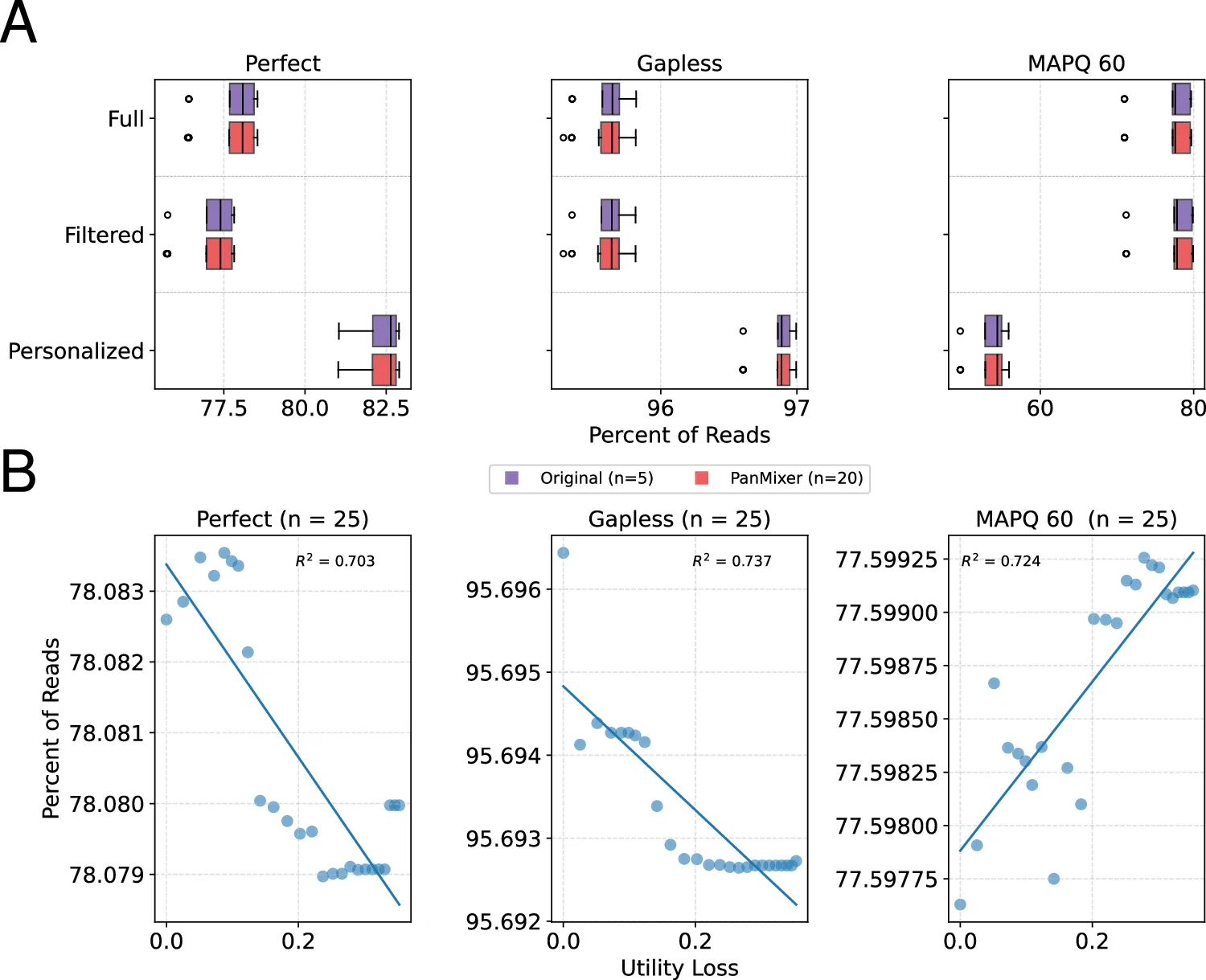
Downstream utility analysis for individuals outside of the pangenome reference. **A** Percent of reads mapped exactly, gaplessly, or with MAPQ 60 on pangenome graphs generated by PanMixer and the original graph, evaluated across three graph constructions: the full pangenome, an allele-frequency-filtered pangenome (variants with alternative allele frequency < 10% removed), and a personalized pangenome^35^. In all box plots, the center line is the median, box bounds are the 25th and 75th percentiles, whiskers extend to 1.5 × IQR, and points beyond are plotted as outliers; minima and maxima are the outermost plotted points. We aligned 1000 Genomes individuals from five populations (EUR, EAS, AMR, SAS, AFR) against four PanMixer graphs, each obfuscating one target individual (HG00438, HG00733, HG02145, HG03492) at a maximum privacy risk of *ϵ*_private_ = 0.002, preventing linkage to the original. For each construction, PanMixer’s mapping rates closely track those of the corresponding original graph, demonstrating that obfuscation preserves downstream alignment quality across all three pangenome regimes. Alignments were performed using vg giraffe^5^ (v1.68.0), and mapping statistics were obtained with vg stats. **B** Correlation between utility loss and percent of perfectly aligned, gapless, and MAPQ 60 reads for the full pangenome with target individual HG00733 obfuscated at various utility loss levels. Utility loss correlated moderately with perfect alignments (*R*^2^ = 0.703), strongly with gapless alignments (*R*^2^ = 0.737), and strongly with MAPQ 60 alignments (*R*^2^ = 0.724).

Figure 5A reports the percentage of perfectly aligned reads (all bases matched), gaplessly aligned reads (matched without any indels), and reads with a mapping quality (MAPQ) of 60^36^. Across all three metrics and all three constructions, the distributions of mapping rates from PanMixer graphs closely overlap those of the corresponding original graphs: on the full graph, perfectly aligned reads averaged 77.82% (vs. 77.83% for the baseline), gapless reads 95.61% (vs. 95.62%), and reads with a MAPQ of 60 77.01% (vs. 77.01%).

We further empirically showed that our utility definition is also correlated with correctly mapped read percentages (Fig. 5B). We found that utility loss correlates moderately with the percent of perfectly aligned reads (*R*^2^ = 0.703), gapless aligned reads (*R*^2^ = 0.737), and reads with a MAPQ of 60 (*R*^2^ = 0.724).

#### Scalability of Privacy Preservation

To assess the scalability of PanMixer, we evaluated downstream utility metrics on pangenome graphs with varying numbers of target individuals (*n* = 1 to *n* = 40). We observed that utility loss generally scales linearly with the size of the protected cohort, with notable deviations at higher counts (Supplementary Fig. S6). Specifically, allele frequency divergence (Wasserstein distance) and gapless read alignment exhibited consistent linear degradation. In contrast, both LD loss and the percentage of perfectly aligned reads showed a steady decline that accelerated when nearly every individual was obfuscated (*n* = 40).

High-quality mapping rates (MAPQ 60) increased, suggesting that decreasing graph diversity through obfuscation removes the subtle sequence variations that the target individuals provided. Through the obfuscation of these unique “features," the process simultaneously reduces the ability for an attacker to link an individual and trends the graph toward a more linear representation (such as GRCh38), which has been shown to have higher mapping rates than pangenome graphs^35^. Ultimately, these results demonstrate that the cumulative utility loss of privacy is predictable and does not impose a prohibitive burden as the protected cohort size increases.

#### Performance of PanMixer

Finally, we measured the computational efficiency and scalability of PanMixer by evaluating its performance on the HPRC v1 (*n* = 44) and v2 (*n* = 231) Minigraph-Cactus graphs (Table 1). Although the v2 cohort is approximately five times larger than v1, the obfuscation process took nearly 16 times as long. This disproportionate increase is due to the HMM sampling and haplotype probability estimation, which scale quadratically with the number of haplotypes in the graph.

**Table 1:** Wall-clock time and peak memory to obfuscate a single subject in the HPRC v1 (44 total subjects) and HPRC v2 (231 total subjects) Minigraph-Cactus pangenome graphs

	HPRC v1 (44)		HPRC v2 (231)	
	Time	Memory	Time	Memory
Obfuscation (per subject)	5 min	14 GB	78 min	142 GB

All 22 chromosomes are run in parallel; the reported time and memory are from the longest-running chromosome. Benchmarks were run on an HPC cluster with an AMD EPYC 9654 96-core processor and 735 GB RAM per node.

## Discussion

In this paper, we present PanMixer, the first system to address privacy in pangenome reference releases. PanMixer obfuscates haplotype paths for selected individuals, enabling participation in the pangenome reference with lower risk of re-identification attacks and membership inference attacks. We showed that PanMixer can substantially reduce the risk of linking an individual’s genome to external databases under state-of-the-art attacks^26^, while preserving the utility of the reference for downstream analyses such as allele frequency estimation, LD analysis, and read mapping.

Unlike exclusion-based strategies or approaches such as removing all unique nodes^34^, PanMixer balances privacy and utility by solving a constrained optimization problem. This generates a privacy-utility tradeoff curve that allows informed choices about the level of privacy protection. Our results demonstrate that obfuscated haplotype paths preserve allele frequency spectra, LD decay vectors, and read mapping quality more effectively than simply excluding those unique DNA sequences.

We also considered Differential Privacy (DP) based approaches, which typically safeguard privacy by injecting statistical noise into the data. While DP is the gold standard for tabular data, applying it to pangenome graphs introduces a critical trade-off between privacy and biological utility. Standard DP mechanisms would require perturbing edges or introducing dummy paths, which breaks the long-range LD structure essential for downstream tasks such as variant calling and read mapping. Recent work has noted that applying DP to pangenome generation often yields sub-optimal results, as the noise required to satisfy privacy bounds destroys the structural integrity of the genome assemblies^34^. Therefore, PanMixer prioritizes the maintenance of topological validity by obfuscating path identifiability through probabilistic switching rather than noise injection, ensuring that the released graph remains biologically useful.

Crucially, the privacy guarantees provided by PanMixer are empirical and address realistic re-identification and membership inference attacks. It is possible that, with additional genomic information or knowledge of the parameters and obfuscation process itself, an attacker could perform stronger attacks than those PanMixer protects against. For large-scale, population-level references where identifiable haplotypes often discourage participation, PanMixer offers a practical solution for improving privacy by reducing both re-identification and membership inference risk. Providing formal, worst-case guarantees against membership inference remains a distinct and important direction for future work.

Similarly, while a closed-form analytical expression characterizing the global privacy-utility trade-off would be theoretically appealing, the heterogeneity of genomic data makes such a derivation intractable without imposing unrealistic simplifying assumptions. In PanMixer, privacy risk and utility loss are defined analytically at the local level–specifically, the removed privacy risk for a block is $-\log p({h}_{{b}_{j}})$, but the global trade-off is governed by the joint distribution of haplotype probabilities and variant supports across the entire genome. Consequently, the relationship between *ϵ* and *η* is determined by a discrete optimization problem over blocks with nonuniform costs and values, which does not admit a simple universal function. Instead, PanMixer provides a precise, data-driven mapping: for any specific pangenome graph, our knapsack formulation computes the exact minimal utility loss required to achieve a given privacy budget *ϵ*. This approach avoids the inaccuracies of loose bounding while capturing the complex, data-dependent structure of real pangenome variation.

Turning to the practical application of our framework, for curators releasing future pangenome reference graphs, we provide the tools to compute the privacy risk threshold *ϵ*_private_, the point at which linking attacks are no longer successful for any target individual. Importantly, our formulation of privacy risk is data-independent and generalizes across individuals, populations, and pangenome graphs. This enables *ϵ*_private_ to be computed for any pangenome graph and any external database, giving curators a principled and reproducible way to determine when the release of a reference is resilient to re-identification attacks.

It is important to note that the specific privacy threshold (*ϵ*_private_) reported in this study is empirical and dependent on the assumed attacker model, specifically the external reference database (1000 Genomes) and the attack strategy employed. A stronger adversary with access to a larger or more genetically similar reference panel might require a stricter privacy threshold (a lower *ϵ*) to prevent linkage. However, PanMixer is designed not to enforce a universal privacy constant, but to provide a flexible, attacker-agnostic framework. By quantifying privacy via Pointwise Mutual Information (PMI), a metric that captures the fundamental statistical dependence exploitable by linkage or reconstruction attacks, PanMixer allows data owners to simulate various threat scenarios. This shifts privacy assessment from a one-shot empirical claim to a configurable decision process: data owners can explicitly evaluate privacy-utility tradeoffs for the specific threat model they consider relevant to their population and release context, and select an operating point accordingly.

We acknowledge that PanMixer relies on accurate population allele frequency and LD estimates to quantify privacy risk. These inputs may currently be less precise for underrepresented populations or novel variants. However, as pangenome references expand in size and diversity, these estimates will naturally improve. This growth does not impose a computational bottleneck, as PanMixer scales linearly with the number of LD blocks and variants, independent of the total reference cohort size. Future work can build on this framework by jointly protecting multiple individuals at scale, optimizing for additional downstream analyses, and deriving analytical bounds on the privacy-utility trade-off under simplified assumptions.

By addressing privacy directly, PanMixer makes pangenome references more inclusive, secure, and equitable. Lowering the risks of participation can encourage contributions from historically underrepresented populations, broadening the diversity of future genomic resources. In this way, PanMixer not only strengthens the technical foundations of pangenome sharing but also helps ensure that the benefits of genomics are distributed more fairly across all communities.

In practice, obfuscation would be applied only with participant consent, and consent forms should explicitly communicate that haplotype paths may be algorithmically modified prior to release to reduce re-identification risk, aligning with existing privacy-preserving data sharing practices while offering participants greater control over how their genomic data are represented.

## Methods

The PanMixer pipeline has three major steps: preprocessing, obfuscation, and optional downstream analysis steps. Preprocessing prepares the graph by splitting it into blocks, obfuscation alters haplotype paths of target individuals using probabilistic models, and downstream analysis evaluates the privacy and utility trade-offs.

### Preprocessing

Pangenome graphs are often represented in Graphical Fragment Assembly (GFA) files^37^, which specify the DNA sequence of each node, the set of possible edges between nodes, and contig paths as ordered node traversals with orientation. These GFAs are powerful and descriptive of the complex graph substructures that can exist inside of pangenomes supporting inversions, loops, etc. From a privacy perspective, their very lack of structure, the feature that makes them so descriptive, also makes them difficult to safeguard against attacks. We opt to use a more systematic representation, such as a Variant Call File (VCF) with nested variants included (produced using tools such as vg deconstruct^1^). Tools like PanGenie^6^ also use this representation, often simplifying further by removing nested variants unless they are on alleles with large reference lengths (>100 kb)^38^. However, we do not remove any variants to preserve some of the complex structures in the graph. We evaluated PanMixer using the Pangenome Graph Builder (PGGB)^8^ reference GFA from the initial human pangenome draft^1^, running the pipeline on all autosomal chromosomes. Variants were extracted using vg deconstruct (v1.36.0) with the -a flag to retain all nested variants, using GRCh38 as the backbone reference.

#### Variant annotations and LD block computation

Let *V* denote all variants and *V*_0_ the set of top-level variants (variants on the GRCh38 haplotype path). Variants are categorized as follows:

• *V*_1000G_ ⊂ *V*_0_: Established variants from the Phase 3 phased 1000 Genomes dataset^25^, used as trusted anchor points.

• *V*_PG_: Variants identified by variant calling of all 1000 Genomes Phase 3 individuals against the draft human pangenome using the Minigraph-Cactus graph.

• *V*_SNPs_ ⊂ *V*_0_: Subset of variants along the GRCh38 reference backbone that are SNPs.

• *V*_novel_ = *V*⧹(*V*_1000G_ ∪ *V*_PG_): Novel variants not represented in existing databases (e.g., 1000 Genomes callset).

Each haplotype block path can be defined using the variants it traverses, as we use a variant-centric view of the pangenome. LD blocks within the pangenome graph were computed using genotypes from the 1000 Genomes Phase 3 variants *V*_1000G_ set. These blocks were computed using plink (v.1.9) with the flag --blocks to compute LD block boundaries^39,40^. We grouped variants within the pangenome graph into LD blocks from the boundaries computed by Plink. Variants outside identified LD blocks were treated as individual blocks, and variants not in *V*_1000G_ but within the boundaries of an LD block were considered part of that LD block.

### Obfuscation

Without loss of generality, we describe the algorithm for obfuscating a single individual inside the pangenome graph. This algorithm can be repeated for any other target individuals inside the pangenome graph. Each individual genome inside the pangenome graph has two haplotype paths, which we obfuscate at the same time. For each LD block, we define two obfuscation moves for both haplotype blocks. We budget utility loss by the sum of the obfuscation moves taken on both haplotype paths, and perform all downstream analysis at the genome level.

In summary, for each LD block *j*, we find two obfuscation moves that replace the existing haplotype block path. For each move, (1) we sample a probable new haplotype block, (2) we compute the removed privacy *ϵ*_*j*_, (3) and we compute the utility loss of taking this obfuscation move *η*_*j*_.

#### Sampling new haplotype block paths

We sample a probable haplotype block path different from the existing haplotype block path of the target individual. This will form a possible move for the obfuscation algorithm to potentially pick. The sampling algorithm is dependent on the number of top-level SNPs that exist inside the LD block. If 2 or more exist, enough SNPs exist to build an HMM^23^, and we can synthesize more accurate new haplotype blocks. If only one top-level SNP exists, we do not have enough useful information to build an HMM. Instead, we sample a new haplotype block path using population allele frequencies.

#### Sampling using population allele frequencies

For LD blocks containing one or no top-level SNPs, we sample alleles directly using population allele frequencies. AF sources depend on the variant type:

• For variants in *V*_1000*G*_, frequencies are taken from the 1000 Genomes dataset.

• For variants in *V*_*PG*_⧹*V*_1000*G*_, frequencies are taken from the PanGenie callset.

• For novel variants not present in either dataset, frequencies are estimated from the pangenome graph itself.

When sampling nested variants, we proceed hierarchically, first sampling from the top-level variant, then sampling nested variants conditional on that choice. This maintains structural consistency in the haplotype path.

#### Sampling a new haplotype block path using an HMM

**Fig. 6:**
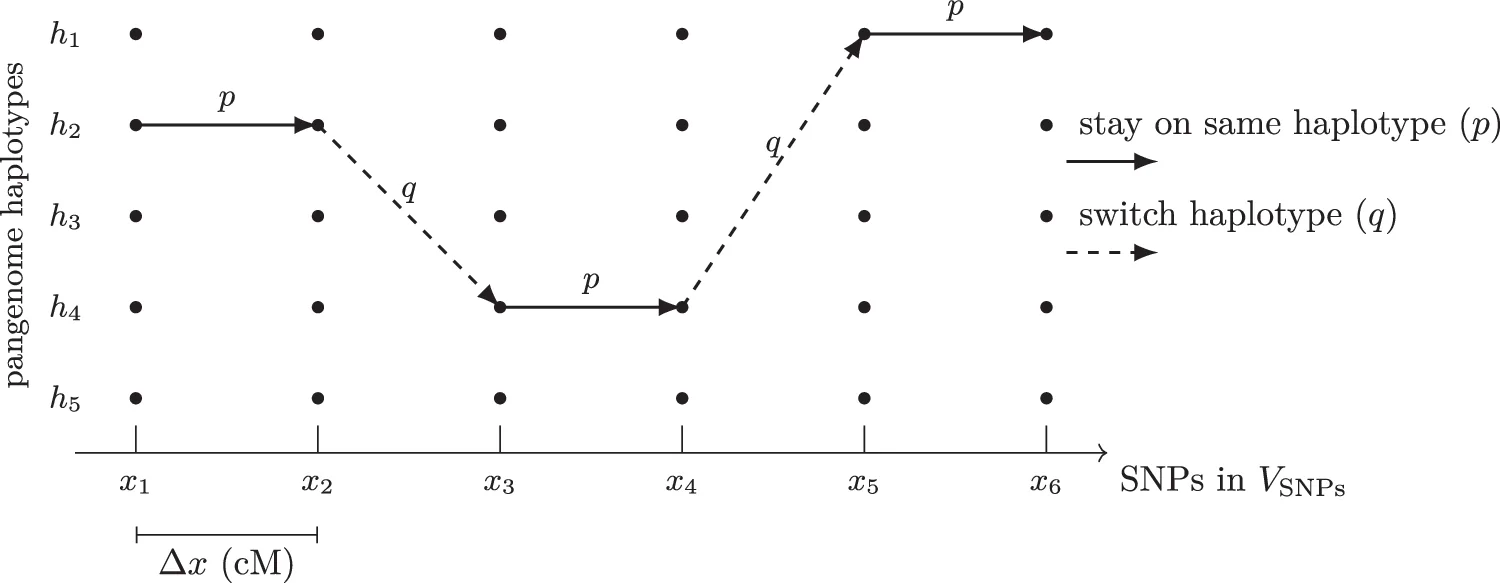
Schematic of the haplotype sampling process using a HMM modeled from Li-Stephens^23^ and PanGenie^6^. PanMixer picks a random starting haplotype, and probabilistically either stays (solid arrow) or switches (dashed arrow and represents a recombination event). The HMM uses SNPs from *x*_*j*_ ∈ *V*_snps_. Model parameters: effective population size *N*_*e*_ = 10,000; standard recombination rate *r* = 1.26 ^43^; distance between SNPs Δ*x* (centiMorgans); *d* = Δ*x* ⋅ *N*_*e*_ ⋅ *r*; stay probability $p={e}^{-\frac{d}{n}}+\frac{1-{e}^{-\frac{d}{n}}}{n}$; switch probability $q=\frac{1-{e}^{-\frac{d}{n}}}{n}$.

When at least two top-level SNPs are present in an LD block, we construct a Li-Stephens HMM^23^ over the donor haplotypes in the pangenome graph (excluding haplotypes belonging to target individuals). The HMM is defined only on top-level SNPs and *V*_SNPs_, so the resulting sampled haplotype path consists of alleles at those positions (see Fig. 6 for details on the HMM model and parameters used).

To incorporate additional complexity, we attach non-top-level variants to the nearest top-level SNP. After sampling a donor path with the HMM, we inherit the alleles of these attached variants from the same donor haplotype used at the neighboring top-level SNP.

The final sampled haplotype block, therefore, combines top-level SNPs chosen via the HMM with attached nested or complex variants propagated from the corresponding donor haplotypes, yielding a structurally consistent haplotype path.

#### Computing privacy risk

While PanMixer could directly minimize privacy risk, it instead formulates the optimization problem in terms of maximizing removed privacy risk, which is mathematically equivalent. This choice is purely a matter of convention, as optimization frameworks such as the knapsack problem are more naturally expressed as maximization problems.

For an LD block *b*_*j*_, the removed privacy risk is given directly by Supplementary Lemma 2 as ${\epsilon }_{j}=-\log p({h}_{{b}_{j}})$, where $p({h}_{{b}_{j}})$ is the population probability of the haplotype path ${h}_{{b}_{j}}$.

**Single top level SNP (or none)** If the LD block contains at most one top-level SNP, we compute $p({h}_{{b}_{j}})$ as the product of allele frequencies across all variants *v* in the block: $p({h}_{{b}_{j}})={\prod }_{v\in {b}_{j}}{f}_{v}({a}_{v}),$ where *a*_*v*_ is the allele of haplotype ${h}_{{b}_{j}}$ at variant *v* and *f*_*v*_( ⋅ ) denotes its population frequency. Allele frequencies are obtained as follows:

• For variants in *V*_1000*G*_, frequencies are taken from the 1000 Genomes dataset.

• For variants in *V*_*PG*_⧹*V*_1000*G*_, frequencies are taken from the PanGenie callset.

• For novel variants not present in either dataset, frequencies are estimated conservatively: the numerator is the number of pangenome haplotypes carrying the allele, and the denominator is the number of 1000 Genomes haplotypes plus the number of pangenome haplotypes not represented in 1000 Genomes. This approach ensures that variants rare enough to be absent from standard callsets, but present in the pangenome graph, are assigned a frequency that reflects their rarity relative to the combined reference population.

For nested variants, the probability is approximated as the product of the population frequency of the parent allele and the marginal frequency of the nested allele. Formally, for a nested variant *v*_nested_ within a parent variant *v*_parent_, we write $\tilde{p}({a}_{{v}_{{{\rm{nested}}}}})\,\approx \,p({a}_{{v}_{{{\rm{parent}}}}})\cdot p({a}_{{v}_{{{\rm{nested}}}}})$, which corresponds to assuming conditional independence of the nested allele given its parent. In practice, this means that the contribution of a nested allele is only counted when its parent allele is present, but the nested AF itself is taken from population-level estimates (1000 Genomes, PanGenie, or the graph). While this simplification may not capture true correlations between nested variants and their parents, it provides an approximation in the absence of large-scale conditional frequency data.

**Multiple top level SNPs** If the LD block contains more than one top-level SNP, we can incorporate LD information in computing $p({h}_{{b}_{j}})$. In this case, we compute $p({h}_{{b}_{j}})$ using the Li-Stephens HMM that we use for the sampling process. The haplotype probability can be evaluated with the forward algorithm, which estimates the total probability of the observed haplotype path ${h}_{{b}_{j}}$ using the top-level SNPs in *b*_*j*_^41^.

#### Computing utility loss

The utility loss of an obfuscation move on a block *b*_*j*_ is ${\eta }_{j}={\sum }_{v\in \Delta }\frac{1}{\,{\rm{support}}\,(v)}$ where Δ is the set of variants altered by the move, and support(*v*) is the number of haplotypes traversing variant *v*. The utility loss treats nested and regular variants the same as the support only counts the number of present haplotypes ignoring missing and skipped haplotypes.

#### Obfuscating target individual with knapsack optimization

For all *k* LD blocks, from the process above, we have 2 obfuscation moves for each LD block and the reduced privacy risk *ϵ*_*j*_ and utility loss *η*_*j*_ for each move.

We define a binary decision vector on whether to take the move or not as **x** ∈ {0, 1}^*k*^. Our realized removed privacy risk and utility loss is a dot product **x** ⋅ *ϵ* and **x** ⋅ *η*.

We then fix the utility loss as Δ*U*, and solve:

4 $${\mathop{\rm{maximize}}\limits_{{{\bf{x}}}\in {\{0,1\}}^{k}}}\,\,\rho ({{\bf{x}}})=\sum _{j=1}^{k}{\epsilon }_{j}{x}_{j}\,\,\,{\rm{subject \,to}}\,\,\, \sum _{j=1}^{k}{\eta }_{j}{x}_{j}\le \Delta U$$

In practice, we utilize the linear optimization solver from OR-Tools^24^ and relax the binary constraint on **x**, rounding to 0 or 1 after the computation has completed. All results produced in this paper were from optimal solutions as reported by OR-Tools. The optimization happened on a per-chromosome level for efficiency purposes.

### Optional downstream analysis

To evaluate the privacy and utility of the obfuscated pangenome graphs, we performed several downstream utility analyses, detailed below.

#### Relationship between privacy risk and linkage attacks

We evaluated whether target individuals could be empirically linked to external genomic databases (with potentially sensitive attributes) using a privacy attack similar to that described in^26–29^. Specifically, we attempted to link target individuals to entries in the 1000 Genomes dataset by computing a genotype linkage score *L* between each individual *g*_*i*_ in the 1000 Genomes dataset and a target individual ${g}_{\,{\rm{target}}\,}^{*}$.

Although both the 1000 Genomes dataset and the pangenome data are phased, we found that matching at the genotype level was the most effective attack strategy, outperforming haplotype-level matching and unweighted matching (Supplementary Fig. S7).

The genotype linkage score is defined as:

5 $$L({g}_{i},{g}_{\,{\rm{target}}\,}^{*})=-\sum_{j\in {{\mathcal{S}}}({g}_{i},{g}_{\,{\rm{target}}\,}^{*})}\log {f}_{j}$$

where: ${{\mathcal{S}}}({g}_{i},{g}_{\,{\rm{target}}\,}^{*})$ is the set of loci where the genotypes of *g*_*i*_ and ${g}_{\,{\rm{target}}\,}^{*}$ are identical, and *f*_*j*_ is the population frequency of the genotype at locus *j*.

This scoring scheme emphasizes rare allele matches, which are more informative for uniquely identifying individuals. An individual is considered successfully linked if the linkage score between the obfuscated genome and the original genome is greater than or equal to all scores with other database entries:

6 $$L({g}_{{{\rm{target}}}},{g}_{{{\rm{target}}}}^{*})\ge L({g}_{i},{g}_{{\rm{target}}\,}^{*})\quad \forall i.$$

We define the gap score as the difference between the linkage score between the obfuscated genome and the original genome and the linkage score between the obfuscated genome and all other database entries:

7 $$\,{\rm{Gap Score}}({g}_{{{\rm{target}}}})=L({g}_{{{\rm{target}}}},{g}_{{{\rm{target}}}}^{*})-{\mathop{\max }\limits_{i\ne {\rm{target}}}}L({g}_{i},{g}_{{\rm{target}}\,}^{*}).$$

Finally, we determine *ϵ*_private_, the privacy risk threshold at which all target individuals can no longer be linked, by systematically obfuscating each individual across decreasing *ϵ* values until the gap score falls below zero for every individual in the pangenome.

### Reconstruction attacks

We simulated a reconstruction attack by modeling an adversary attempting to recover the original haplotype paths from the obfuscated haplotypes. The attacker was assumed to have access to the 1000 Genomes dataset as a reference panel.

For target individuals within our pangenome graph, we first extracted SNPs overlapping with those in the 1000 Genomes dataset. Using only the genotypes at these overlapping SNPs, we ran Beagle (v5.5)^30^ with default parameters for genotype refinement, with the 1000 Genomes dataset (excluding the target individuals) as the reference panel. We then compared the refined genotypes both to the original genotypes and to a baseline reconstruction obtained by selecting the most frequent alleles from the 1000 Genomes dataset.

#### Relationship between utility loss and allele frequencies

We modeled each allele at every variant in the pangenome graph as a Bernoulli random variable, and measured the changes in AFs using the Wasserstein divergence between the original and obfuscated allele distributions. For alleles modeled as a Bernoulli random variable, the Wasserstein divergence is two times the mean absolute difference between AFs before and after obfuscation. The factor of two arises because each diploid individual carries two alleles per variant. This approach follows the method of^42^, but extends it from the minor allele to all alleles.

Formally, let *p*_*j*_ and *q*_*j*_ denote the allele frequencies at site *j* in the original and graphs with obfuscated individuals, respectively. The AF divergence is defined as:

8 $${U}_{{{\rm{AF}}}}=2\cdot \frac{1}{m}\sum _{j=1}^{m}\left\vert {p}_{j}-{q}_{j}\right\vert,$$

where *m* is the total number of variant sites.

#### Relationship between utility loss and linkage disequilibrium

We assess changes in LD structure by comparing the LD matrices of the pangenome graphs with obfuscated target individuals to those of the original graph. LD matrices are computed in sliding windows of 5 kb. Let **d**_original_ and **d**_obf_ denote the LD matrices for the original and obfuscated graphs, respectively. The change in LD is defined as the L1 norm between these two matrices:

9 $${U}_{{{\rm{LD}}}}=| | {{{\bf{d}}}}_{{{\rm{obf}}}}-{{{\bf{d}}}}_{{{\rm{original}}}}| {| }_{1}.$$

#### Relationship between utility loss and read mapping

We evaluated read mapping performance by aligning sequencing reads from randomly selected 1000 Genomes individuals^25^ to pangenome graphs produced by PanMixer. For each population, we randomly selected one individual who was not included in the construction of the pangenome graph: HG00138 (European), HG00635 (East Asian), HG01112 (American), HG02698 (South Asian), and NA18853 (African).

Chromosome 21 reads for these individuals were extracted from CRAM files obtained from the 1000 Genomes repository. The extracted reads were then aligned with Giraffe^5^ to four chromosome 21 pangenome graphs, each with a target individual from a unique superpopulation, obfuscated until linking was no longer possible. The GAM files produced by Giraffe were analyzed using vg stats (v1.36.0) to find the percent reads aligned perfectly, gapless, and with a MAPQ of 60.

### Statistics & reproducibility

No statistical method was used to predetermine sample size. For all analyses other than the read mapping experiments, we used every individual in the HPRC v1.0 and v2.0 pangenome graphs, with no subsampling. For the read mapping experiments, we randomly selected one 1000 Genomes individual from each superpopulation who was absent from the pangenome graph and aligned their reads to four HPRC v1.0 pangenome graphs, each obfuscating a single randomly selected target individual from a distinct superpopulation.

The experiments were not randomized, except that PanMixer’s obfuscation algorithm includes randomization to sample new haplotype paths. We have included the fixed seed used to sample the obfuscated haplotype paths used in all reported results in the code repository.

### Reporting summary

Further information on research design is available in the Nature Portfolio Reporting Summary linked to this article.

## Supplementary information

Supplementary Information

Reporting Summary

Transparent Peer Review file

## Data Availability

The pangenome graphs (HPRC v1.0 and v2.0) used in this study are available in the Human Pangenomics AWS Open Data registry at https://s3-us-west-2.amazonaws.com/human-pangenomics/index.html. The genotype callset used in this study is available in the Zenodo database under accession code https://doi.org/10.5281/zenodo.7669083. The population reference haplotypes used in this study are available in the 1000 Genomes Project phase 3 release (European Bioinformatics Institute, release 20130502) at https://ftp.1000genomes.ebi.ac.uk/vol1/ftp/release/20130502/. Scripts to download all datasets are provided in the PanMixer GitHub repository (https://github.com/g2lab/panmixer).

## Code availability

The code for PanMixer is available at https://github.com/g2lab/panmixer.
