# Supplementary material for: Privacy-preserving pangenome graphs
Source: Nat Commun. 2026 Sep 14;17:10805. doi: 10.1038/s41467-026-77591-0 (PMC13669366; doi:10.1038/s41467-026-77591-0)
Supplement: Supplementary file 1 [file 41467_2026_77591_MOESM1_ESM.pdf]

Supplementary Materials

This document contains the supplementary materials for the paper *Privacy-Preserving Pangenome Graphs*.

Supplementary Figures

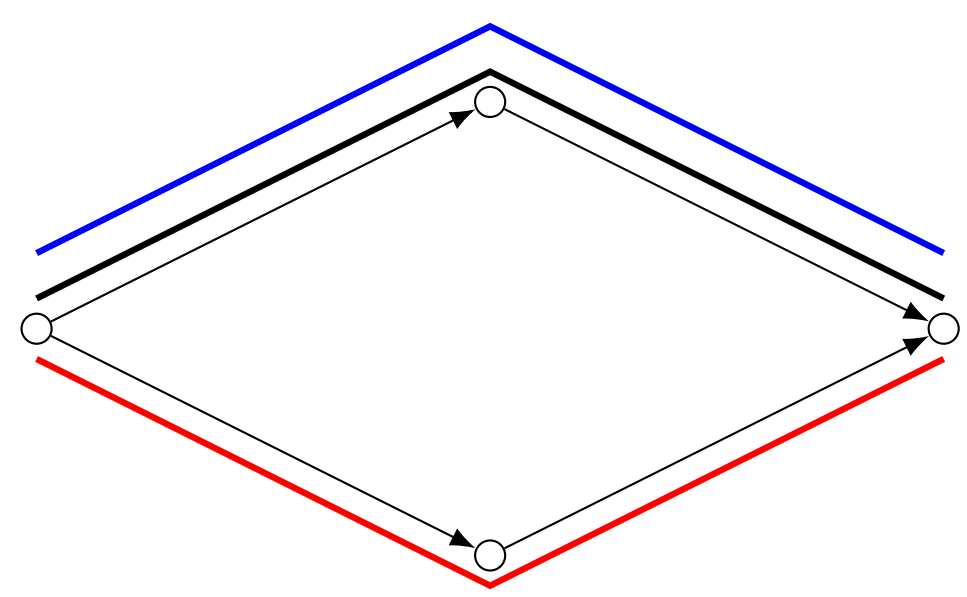

Figure S1: A SNP inside the pangenome graph. The black line represents the linear haplotype reference backbone (such as GRCh38), the blue line represents the target individual's haplotype path, and the red line represents the obfuscated haplotype path.

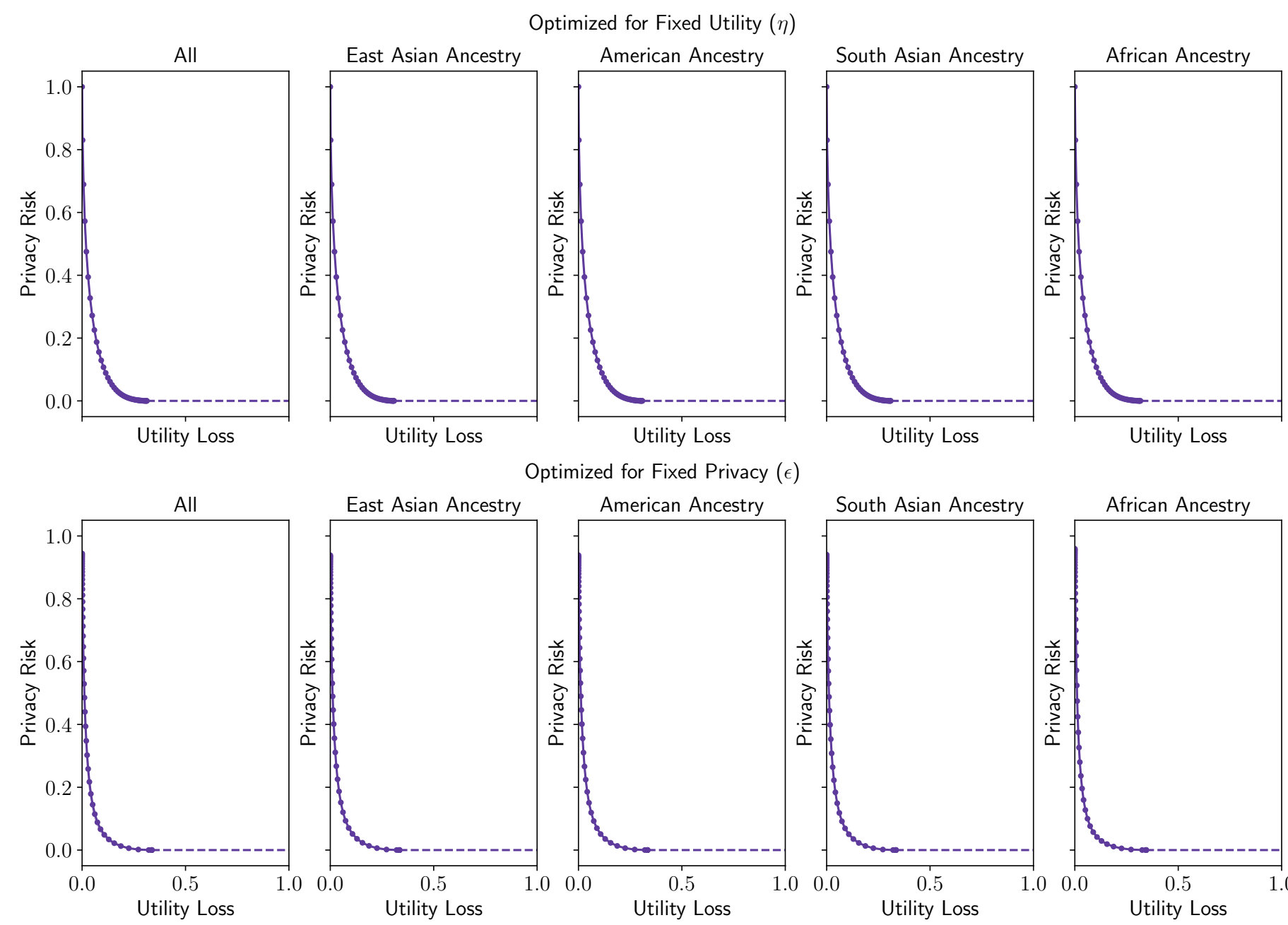

Figure S2: Privacy–utility trade-off across ancestries. Privacy risk (ϵ) achieved by PanMixer across a range of fixed utility-loss levels, and utility loss (η) achieved across a range of fixed privacy-risk levels. Each plot shows results averaged over all individuals ($n = 44$) and stratified by ancestry: East Asian ($n = 4$), American ($n = 16$), South Asian ($n = 1$), and African ($n = 23$).

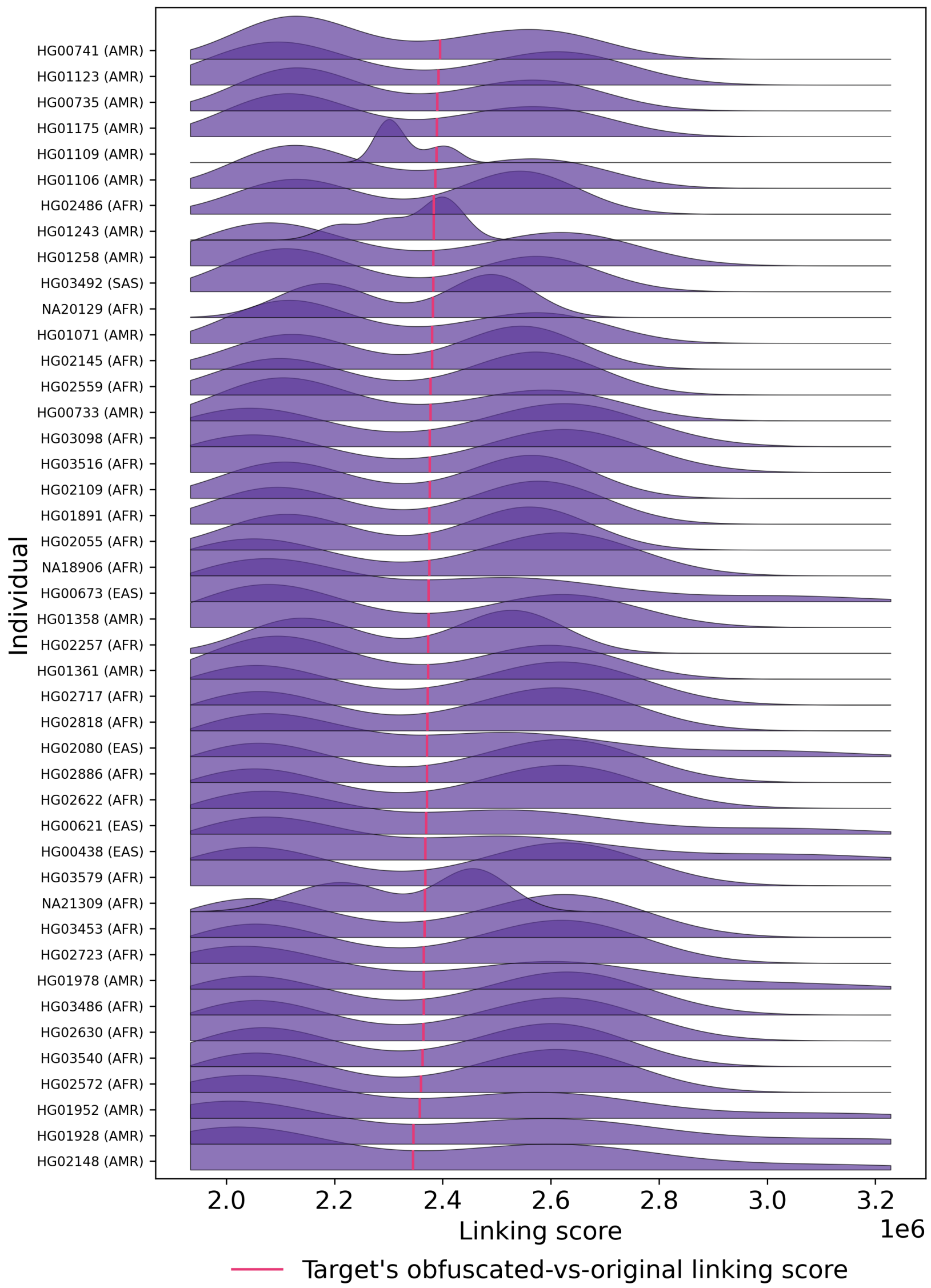

Figure S3: Per-individual distribution of linking scores for an individual's original genome against all other subjects in a pangenome with this original genome obfuscated to $\epsilon_{\text{private}} = 0.002$. For each row, the ridge summarizes the distribution of linking scores. The red line represents the linking score between the original and obfuscated genomes. A red line positioned within the bulk of the linking score distribution suggests limited ability for an attacker to discern whether the target individual was part of the pangenome.

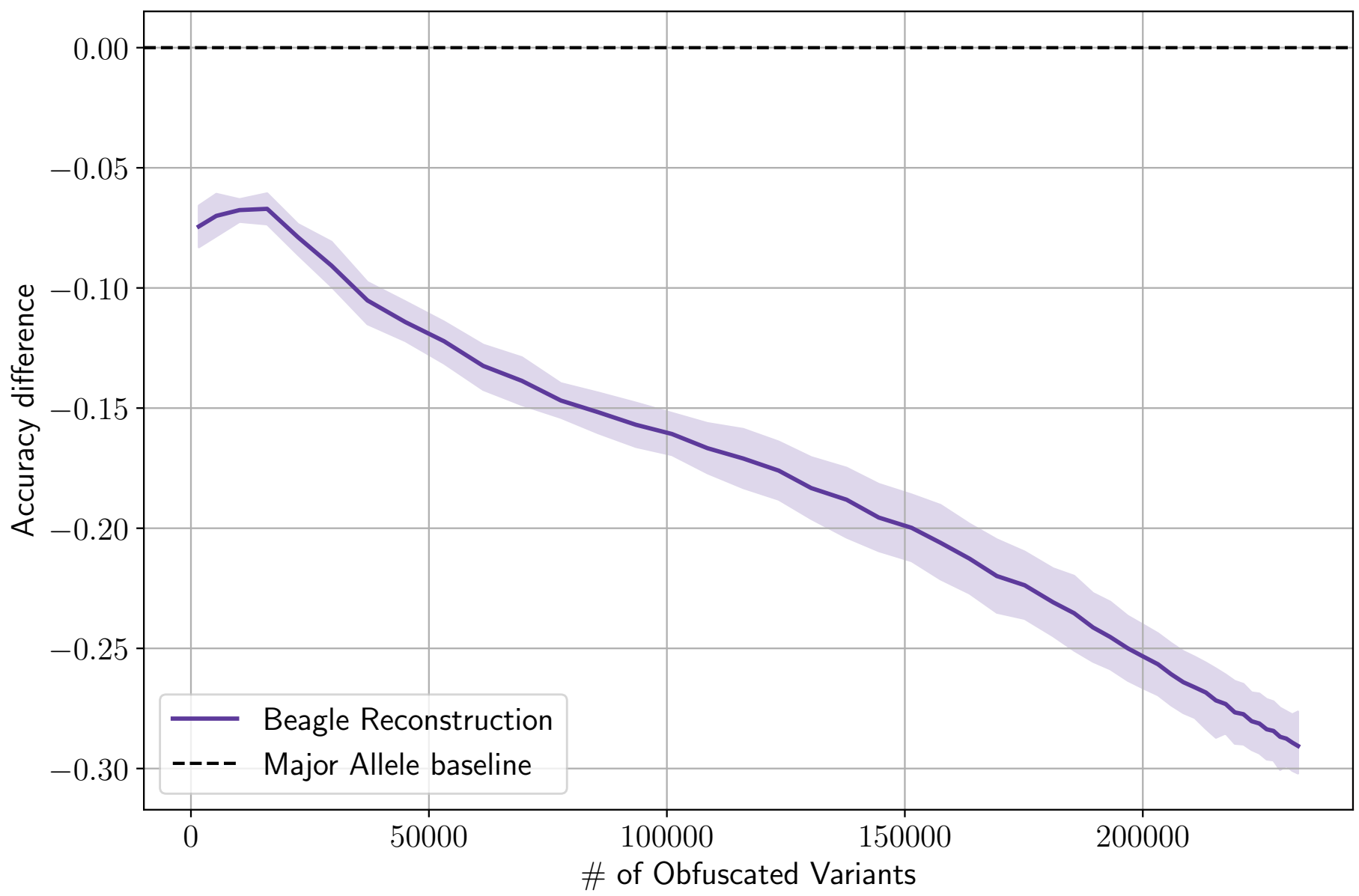

Figure S4: Difference in imputation accuracy between Beagle and a simple major allele baseline across increasing numbers of obfuscated variants. For various privacy loss levels, we compute the mean accuracy difference (Beagle vs. Major Allele). Negative values indicate that Beagle reconstruction performs worse than simply predicting the major allele. Shaded regions highlight uncertainty, and the dashed horizontal line marks parity with the baseline. Results are shown for chromosome 21 reconstructed using Beagle (version 5.5) with the 1000 Genomes genotypes as the reference panel.

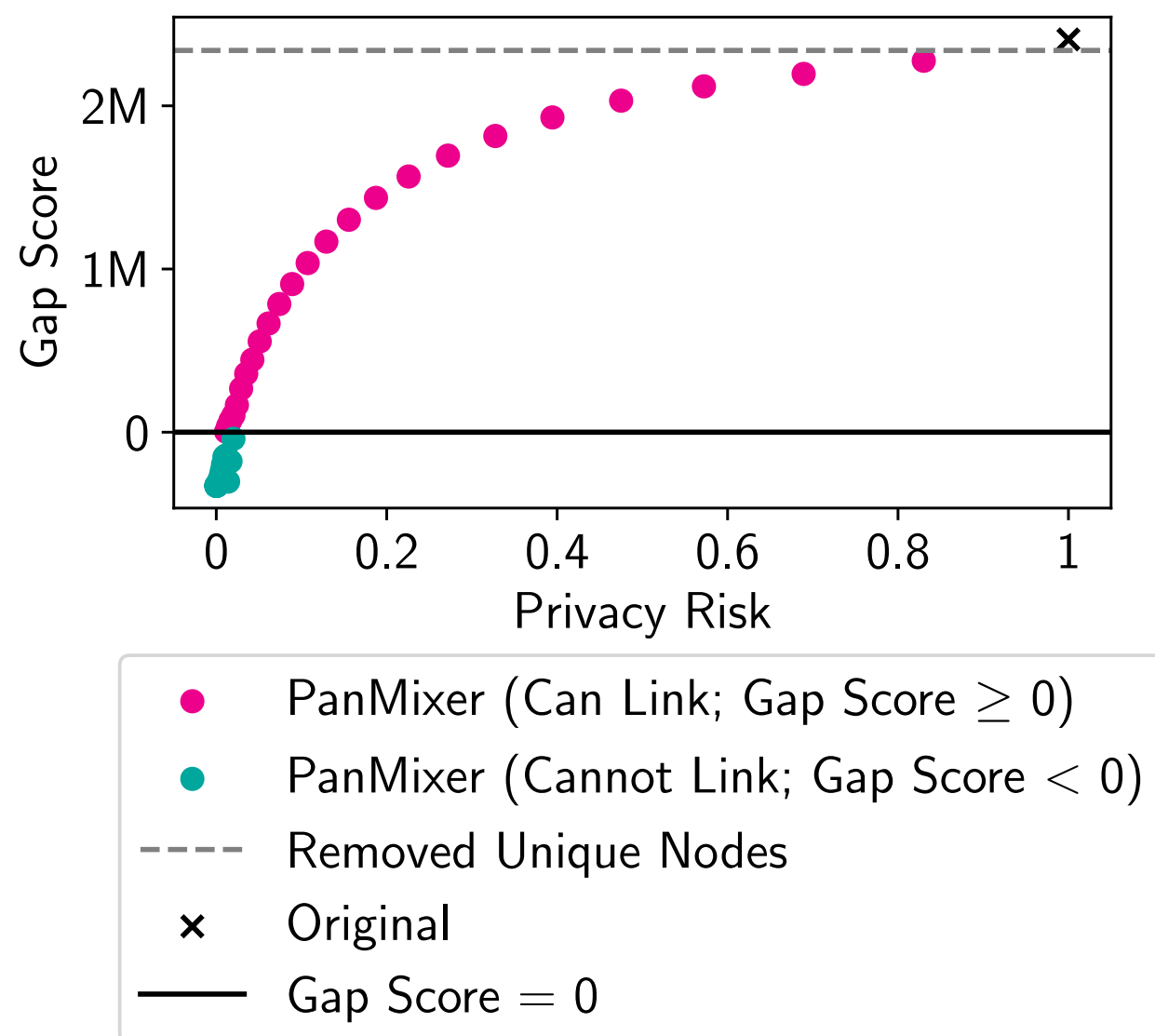

Figure S5: Comparison of re-identification risk across obfuscation strategies. A Gap Score > 0 (above the solid black line) indicates successful re-identification, while a score < 0 implies the attack failed. The "Removed Unique Nodes" baseline (grey dashed line) maintains a high positive Gap Score nearly identical to the Original unobfuscated graph (black cross), demonstrating that removing rare variation alone provides negligible protection against linkage attacks. In contrast, PanMixer (circles) systematically reduces gap scores as the privacy risk decreases. The transition from pink points (can link) to teal points (cannot link) illustrates that PanMixer can successfully drive the Gap Score below zero, rendering the individual unlinkable.

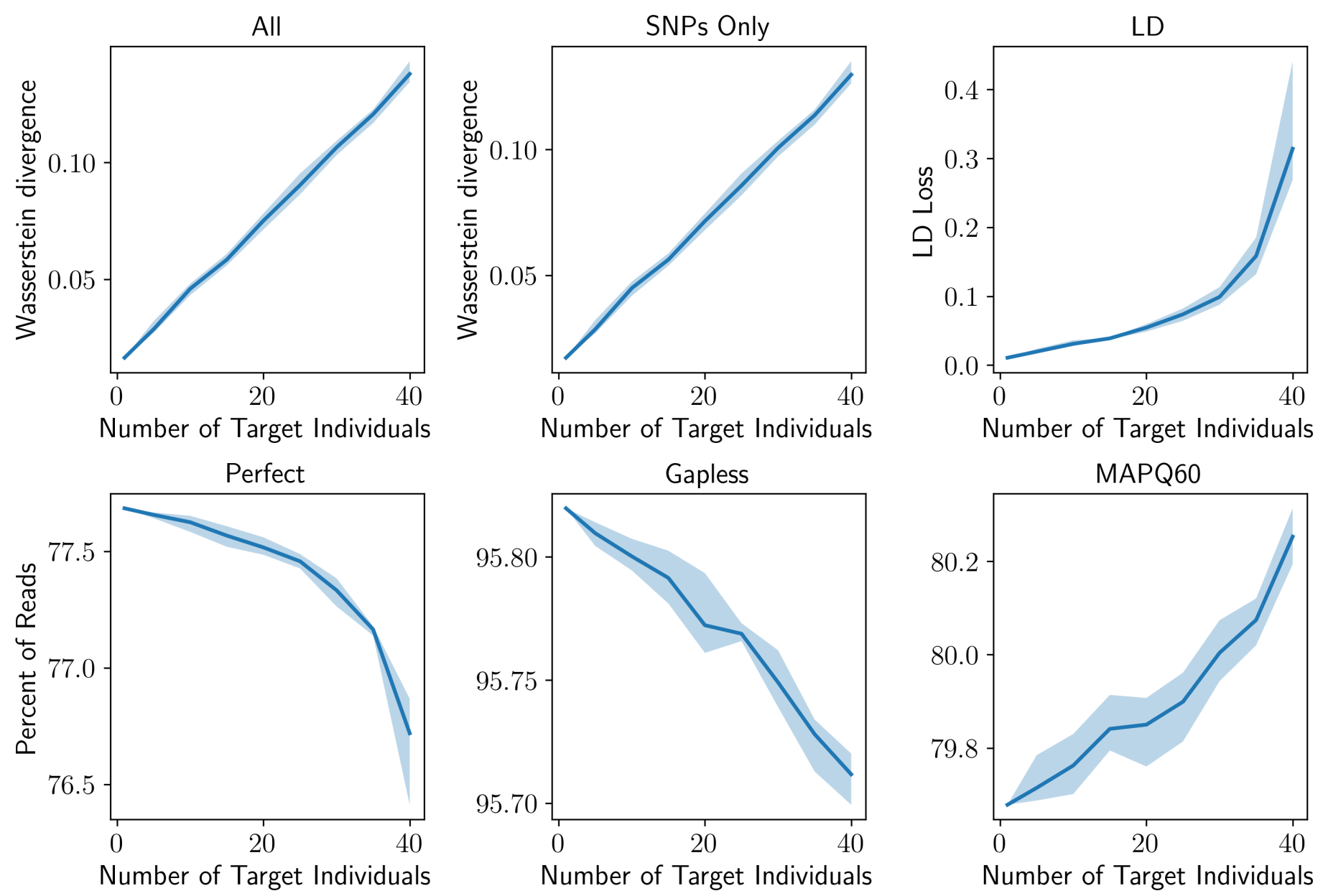

Figure S6: Downstream utility metrics as a function of the number of protected individuals. For each count, we measured metrics across 5 random subsets; the solid line represents the mean and the shaded region indicates the min-max range. The figure displays the impact of simultaneously obfuscating up to 40 target individuals (x-axis) on various genomic utility metrics. Top row: Allele frequency divergence (Wasserstein distance) for all variants and SNPs only, and Linkage Disequilibrium (LD) loss. Bottom row: Read mapping performance, including the percentage of perfectly aligned reads, gapless alignments, and reads with high mapping quality (MAPQ60).

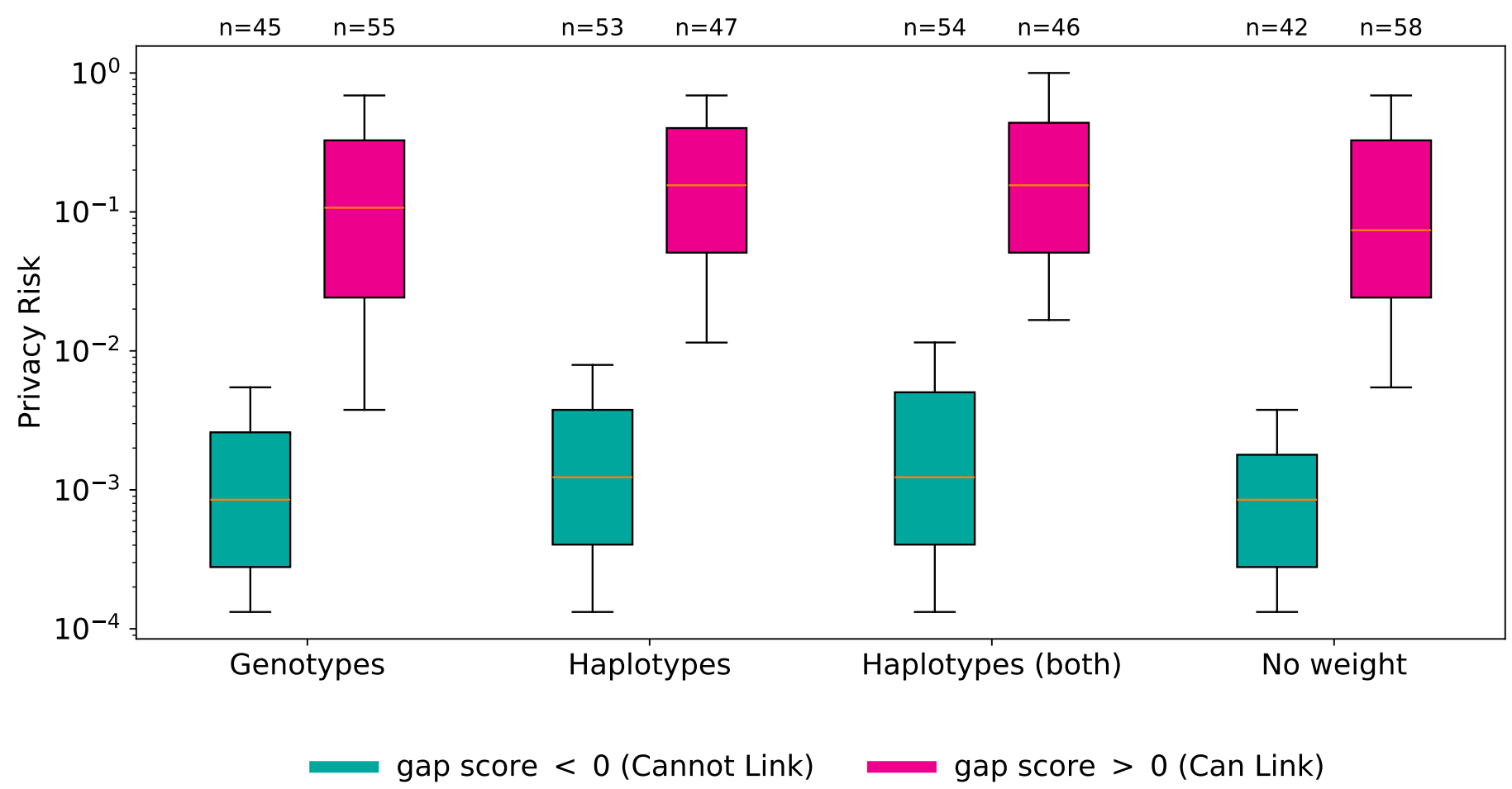

Figure S7: Box plots of privacy risk across all populations for four scoring metrics: *genotypes*, which matches genotypes on the target individual's genome to those in the attack database; *haplotypes*, which matches at the haplotype level rather than the genotype level; *haplotypes (both)*, which considers only sites where both haplotypes are equal and phased identically; and *no weight*, which does not weight shared variants by their rarity. For each metric, two distributions are shown: samples with Gap Score < 0 in cyan (cannot link) and Gap Score > 0 in purple (can link). Genotype-based linking proved most effective, requiring the lowest privacy risk to ensure that the target individual could not be linked.

Supplementary Methods

Tables

Table S1: Mathematical notation

Symbol	Description
Optimization & Trade-off Variables	
ϵ / ϵ_{tot}	Privacy risk, quantified using Pointwise Mutual Information (PMI)
η	Utility loss, measured by Weighted Path Edit Distance (WPED)
x	Binary decision vector ($x \in \{0,1\}^{2k}$) indicating selected obfuscation moves
$\rho(x)$	Removed privacy risk, defined as $\sum \epsilon_j x_j$
$\epsilon_{\text{res}}(x)$	Realized privacy risk after obfuscation ($\epsilon_{\text{tot}} - \rho(x)$)
ΔU	Maximum allowable utility loss (Knapsack capacity)
$\epsilon_{\text{private}}$	Privacy risk threshold where linking attacks fail for all targets
Graph & Haplotype Definitions	
h	The original haplotype path
h^*	The obfuscated haplotype path
b_j	The j-th Linkage Disequilibrium (LD) block
δ_j	An obfuscation move applied to block b_j
$c(\delta_j)$	The cost associated with obfuscation move δ_j
η_j	The utility loss associated with obfuscation move δ_j
ϵ_j	Privacy risk associated with obfuscation move δ_j
V	Set of all variants in the pangenome graph
V_0	Set of top-level variants on the linear reference backbone
V_{SNPs}	Subset of V_0 consisting of Single Nucleotide Polymorphisms
V_{1000G}	Variants established from the 1000 Genomes Phase 3 dataset
support(v)	Number of haplotypes traversing variant v
Metrics & Scoring	
PMI(h, h^*)	Pointwise Mutual Information between original and obfuscated paths
WPED(h, h^*)	Weighted Path Edit Distance between original and obfuscated paths
$L(g, g^*)$	Linkage score between a genome g and obfuscated genome g^*
U_{AF}	Allele Frequency divergence (Wasserstein divergence)
U_{LD}	Change in LD structure (L_1 norm of LD matrix difference)
HMM Parameters (Li-Stephens Model)	
N_e	Effective population size
r	Recombination rate
d	Distance between SNPs in centiMorgans
p	Probability of staying on the same haplotype
q	Probability of switching haplotypes (recombination)

Proofs

Remark 1 (Utility loss is similar to AF)**.** Consider a single SNP (See Supplementary Fig. S1 for an example) inside the pangenome graph on a linear reference backbone such as GRCh38, and the haplotype of an individual taking this reference path. Redirecting the haplotype through the alternate allele changes its AF by

$$L_1(\mathrm{AF}_v, \mathrm{AF}_{v^*}) = \left| \frac{n_a}{\mathrm{support}(v)} - \frac{n_a + 1}{\mathrm{support}(v)} \right| = \frac{1}{\mathrm{support}(v)},$$

where L_1 is the L_1 (absolute difference) loss and n_a is the count of alternate alleles. The utility loss is constant for this move and does not depend on AF.

Lemma 1 (Blockwise additivity of PMI)**.** Let h and h^* be haplotype paths decomposed into k LD blocks $h = (h_{b_1}, \ldots, h_{b_k})$ and $h^* = (h^*_{b_1}, \ldots, h^*_{b_k})$. Assume the following blockwise factorizations hold:

$$p(h) = \prod_{j=1}^{k} p(h_{b_j}), \qquad p(h^*) = \prod_{j=1}^{k} p(h^*_{b_j}), \qquad p(h, h^*) = \prod_{j=1}^{k} p(h_{b_j}, h^*_{b_j}).$$

Then the pointwise mutual information (PMI) decomposes as

$$\mathrm{PMI}(h, h^*) \;=\; \sum_{j=1}^{k} \mathrm{PMI}\big(h_{b_j}, h^*_{b_j}\big).$$

Proof. By definition,

$$\mathrm{PMI}(h, h^*) \;=\; \log \frac{p(h, h^*)}{p(h)\, p(h^*)}.$$

Using the stated factorizations,

$$\begin{aligned}
\mathrm{PMI}(h, h^*) &= \log \frac{\prod_{j=1}^{k} p(h_{b_j}, h^*_{b_j})}{\Big(\prod_{j=1}^{k} p(h_{b_j})\Big)\Big(\prod_{j=1}^{k} p(h^*_{b_j})\Big)} \\
&= \log \prod_{j=1}^{k} \frac{p(h_{b_j}, h^*_{b_j})}{p(h_{b_j})\, p(h^*_{b_j})} \\
&= \sum_{j=1}^{k} \log \frac{p(h_{b_j}, h^*_{b_j})}{p(h_{b_j})\, p(h^*_{b_j})} \\
&= \sum_{j=1}^{k} \mathrm{PMI}\big(h_{b_j}, h^*_{b_j}\big).
\end{aligned}$$

□

Lemma 2 (Removed privacy risk of an obfuscated block)**.** Let h be a haplotype path with LD block decomposition $h = (h_{b_1}, \dots, h_{b_k})$, and let h^* be an obfuscated version of h that differs only at block j^*. Then the removed privacy risk, the reduction in PMI when block j^* is obfuscated, is equal to the self-information of the original block:

$$\mathrm{PMI}(h, h) - \mathrm{PMI}(h, h^*) \;=\; -\log p(h_{b_{j^*}}).$$

Proof. By definition, the removed privacy risk is

$$\mathrm{PMI}(h, h) - \mathrm{PMI}(h, h^*).$$

Applying Lemma 1, we expand both terms blockwise:

$$\mathrm{PMI}(h, h) - \mathrm{PMI}(h, h^*) = \sum_{j=1}^{k} \mathrm{PMI}(h_{b_j}, h_{b_j}) - \sum_{j=1}^{k} \mathrm{PMI}(h_{b_j}, h^*_{b_j}).$$

Since h and h^* differ only at block j^*, all terms cancel except the one at j^*:

$$\mathrm{PMI}(h, h) - \mathrm{PMI}(h, h^*) = \mathrm{PMI}(h_{b_{j^*}}, h_{b_{j^*}}) - \mathrm{PMI}(h_{b_{j^*}}, h^*_{b_{j^*}}).$$

Because the obfuscated block $h^*_{b_{j^*}}$ is sampled independently of $h_{b_{j^*}}$, we have $\mathrm{PMI}(h_{b_{j^*}}, h^*_{b_{j^*}}) = 0$. Thus

$$\mathrm{PMI}(h, h) - \mathrm{PMI}(h, h^*) = \mathrm{PMI}(h_{b_{j^*}}, h_{b_{j^*}}).$$

Finally, expanding the definition,

$$\mathrm{PMI}(h_{b_{j^*}}, h_{b_{j^*}}) = \log \frac{p(h_{b_{j^*}}, h_{b_{j^*}})}{p(h_{b_{j^*}})^2} = \log \frac{p(h_{b_{j^*}})}{p(h_{b_{j^*}})^2} = -\log p(h_{b_{j^*}}),$$

since the joint of a block with itself is its marginal, $p(h_{b_{j^*}}, h_{b_{j^*}}) = p(h_{b_{j^*}})$. □
