# Supplementary material for: Privacy-preserving pangenome graphs
Source: Nat Commun. 2026 Sep 14;17:10805. doi: 10.1038/s41467-026-77591-0 (PMC13669366; doi:10.1038/s41467-026-77591-0)
Supplement: Supplementary file 3 [file 41467_2026_77591_MOESM3_ESM.pdf]

nature portfolio

Peer Review File

Privacy-Preserving Pangenome Graphs

Corresponding Author: Dr Gamze Gürsoy

This file contains all reviewer reports in order by version, followed by all author rebuttals in order by version.

Version 0:

Reviewer comments:

Reviewer #1

(Remarks to the Author)
Blindenbach and colleagues describe an approach for perturbing a pangenome graph to sufficiently disrupt the haplotypes and pangenome graph topology as to make it difficult to impossible to identify which haplotype was contributed by a given participant.

I found the study to be well thought out and the paper to be reasonably well written. I liked the thoughtful use of LD blocks to attempt to preserve linkage structure and avoid linkage attacks. I think the work is an important and thought provoking contribution that very much deserves to be published, however, I have a couple of significant concerns as stated.

Firstly, and probably most significantly, I think the authors should be clearer about what the privacy gains sort by the method actually deliver. In the work the authors say:

"As shown in Fig. 3A, the attacker attempts to link each obfuscated haplotype path to genomes in the 1000 Genomes Project Phase 3 callset [1], using a rarity-weighted matching score that emphasizes shared rare variants, similar to approaches previously used [15, 17, 16, 39]."

This is subtle, but it is important to understand that this is reidentification of a specific haplotype in the pangenome graph. Of course, there is potential privacy value in being able to obfuscate which haplotype belongs to which participant, but this is different from the stricter and perhaps more important question of preventing reidentification of whether an individual contributed to the pangenome. I.e. it seems to me that it is highly likely, based upon using a collection of rare SNPs, that it is possible to determine if an individual contributed their genome to even an obfuscated pangenome. While this might seem like limited knowledge here, for obfuscated pangenomes built to represent specific phenotypes (i.e. those associated with disease) it would seem to be the important question. Of course, as population pangenomes grow very large in the future, perhaps the obfuscation sort by the authors is more important (i.e. merely identifying inclusion in such a large pangenome is perhaps an acceptable risk providing the individual genome can not be precisely identified). I can imagine different scenarios, but I think the distinction between the strict question of “can you identify an individual ’s haplotypes in a pangenome based upon knowledge of their genomes” and “can you identify if an individual contributed to a given pangenome” needs to be discussed properly. The discussion says: “PanMixer obfuscates haplotype paths for selected individuals, enabling participation in the pangenome reference without the loss of privacy.”, which is only true for the stricter, former question. The authors could and should do a much better job in discussing the limitations of the privacy guarantees their work sets forth.

My second big issue with the paper is that the overview of the methods omits some crucial details in the main exposition. Specifically, the details of the haplotype probability calculation p(h) and the joint probability p(h, h’) is not stated. I think it is particularly important to describe these functions in the main text because the paper largely rests on their details - their relationship defines precisely how we are to understand the privacy guarantees presented. I shouldn’t have to go walking through the supplementary material to refer to a lemma to understand this. Similarly, I somewhat get the intuition about the obfuscation moves but I think this could be much clearer - and proper figure illustrating the moves would help. Overall, I think the writers should step back and try to explain the core ideas in the main text more precisely and with sufficient detail.

Smallers nits/comments:

“WPED is conceptually similar to the Weighted Graph Edit Distance (WGED), but instead of measuring

edits between nodes and edges of two graphs, it measures edits between the original pangenome graph and the graph with obfuscated haplotype paths. A low WPED indicates few edits (high utility), whereas a high WPED indicates many edits (low utility)."

This is confusing, because in the prior paragraph you're talking about WPED in terms of individual haplotypes, not the whole pangenome.

The read mapping section is good, although I think it is worth pointing out that mapping to pangenome graphs already either employs selective filtering or "personalized" subsetting of a given pangenome, i.e. it does not surprise me that the obfuscation moves, which seem likely to preserve structure, have only a very small effect on read mapping.

(Remarks on code availability)

Reviewer #3

(Remarks to the Author)
The authors present PanMixer, a framework that selectively obfuscates individual haplotype paths in a pangenome graph in order to reduce re identification risk while preserving the utility of the reference for downstream analyses (allele frequency estimation, linkage disequilibrium (LD) analysis, read mapping, etc.). The privacy utility trade off is cast as a 0-1 knapsack problem: each possible "obfuscation move" on an LD block carries a utility cost (Weighted Path Edit Distance, WPED) and a privacy benefit (point wise mutual information, PMI). The optimal subset of moves is obtained with an integer linear solver (OR Tools). Experiments on the draft human pangenome (47 individuals) demonstrate that (i) linkage based re identification attacks fail once the privacy metric ε falls below a threshold (ε_private ≈ 0.002), (ii) genotype imputation attacks do not recover more information than a simple major allele baseline, and (iii) key downstream tasks show only negligible degradation (AF Wasserstein divergence < 0.01, LD L1 loss < 0.02, read mapping metrics within 0.1 % of the original). The authors argue that PanMixer will enable broader participation, especially from historically under represented populations, in future large scale pangenome projects.

The manuscript tackles an important, timely problem with a solid methodological contribution and a thorough experimental validation. The concerns listed above are primarily about scalability, broader utility validation, and clarification of threat models. Addressing these points will significantly strengthen the paper.

Major comments:
1. The scalability of the proposed method is not sufficiently addressed. In this study, experiments are conducted using haplotypes from only 47 individuals. Provide runtime/memory benchmarks for a realistic scenario (e.g., 350 genomes).

2. Privacy metric sensitivity to allele frequency estimation – PMI relies on population allele frequencies derived from 1000 Genomes, PanGenie, and the graph itself. The impact of miss estimated frequencies (e.g., for under represented groups) on ε is unclear.

3. The authors did not discuss how participants are informed about the obfuscation process or how consent forms should be adapted. Add a brief discussion on consent implications, e.g., whether participants need to be told that their haplotypes will be altered, and how this aligns with existing data sharing policies.

(Remarks on code availability)

Version 1:

Reviewer comments:

Reviewer #1

(Remarks to the Author)
Thanks, the authors have satisfactorily answered all my queries. I recommend publication. Thanks!

(Remarks on code availability)

Reviewer #3

(Remarks to the Author)
The authors have done an excellent job addressing all of my previous concerns. In particular, the inclusion of runtime and memory benchmarks on larger pangenomes, the improved handling of allele frequency estimation, and the added discussion on consent significantly strengthen the manuscript. The revisions demonstrate both technical rigor and thoughtful consideration of practical deployment.
The additional experiments and clarifications enhance the paper's credibility and impact.
I have no further concerns and recommend acceptance.

(Remarks on code availability)

Open Access This Peer Review File is licensed under a Creative Commons Attribution 4.0 International License, which permits use, sharing, adaptation, distribution and reproduction in any medium or format, as long as you give appropriate credit to the original author(s) and the source, provide a link to the Creative Commons license, and indicate if changes were made.
In cases where reviewers are anonymous, credit should be given to 'Anonymous Referee' and the source.
The images or other third party material in this Peer Review File are included in the article's Creative Commons license, unless indicated otherwise in a credit line to the material. If material is not included in the article's Creative Commons license and your intended use is not permitted by statutory regulation or exceeds the permitted use, you will need to obtain permission directly from the copyright holder.
To view a copy of this license, visit https://creativecommons.org/licenses/by/4.0/

Response Letter

We are grateful for the thorough review process and the detailed feedback provided on our manuscript, "**Privacy-Preserving Pangenome Graphs".** The insights and suggestions from the reviews were particularly beneficial and constructive. Below you can find our point-by-point response to your concerns.

Reviewer 1's Strengths

> Blindenbach and colleagues describe an approach for perturbing a pangenome graph to sufficiently disrupt the haplotypes and pangenome graph topology as to make it difficult to impossible to identify which haplotype was contributed by a given participant.
>
> I found the study to be well thought out and the paper to be reasonably well written. I liked the thoughtful use of LD blocks to attempt to preserve linkage structure and avoid linkage attacks. I think the work is an important and thought provoking contribution that very much deserves to be published, however, I have a couple of significant concerns as stated.

We thank the reviewer for their thoughtful, constructive feedback. We have carefully addressed all concerns raised in the detailed response below.

Reviewer 1's Concerns

R1.1: Privacy Clarifications

Reviewer Comment	Firstly, and probably most significantly, I think the authors should be clearer about what the privacy gains sort by the method actually deliver. In the work the authors say:<br><br>"As shown in Fig. 3A, the attacker attempts to link each obfuscated haplotype path to genomes in the 1000 Genomes Project Phase 3 callset [1], using a rarity-weighted matching score that emphasizes shared rare variants, similar to approaches previously used [15, 17, 16, 39]."

	This is subtle, but it is important to understand that this is reidentification of a specific haplotype in the pangenome graph. Of course, there is potential privacy value in being able to obfuscate which haplotype belongs to which participant, but this is different from the stricter and perhaps more important question of preventing reidentification of whether an individual contributed to the pangenome. I.e. it seems to me that it is highly likely, based upon using a collection of rare SNPs, that it is possible to determine if an individual contributed their genome to even an obfuscated pangenome. While this might seem like limited knowledge here, for obfuscated pangenomes built to represent specific phenotypes (i.e. those associated with disease) it would seem to be the important question. Of course, as population pangenomes grow very large in the future, perhaps the obfuscation sort by the authors is more important (i.e. merely identifying inclusion in such a large pangenome is perhaps an acceptable risk providing the individual genome can not be precisely identified). I can imagine different scenarios, but I think the distinction between the strict question of "can you identify an individual 's haplotypes in a pangenome based upon knowledge of their genomes" and "can you identify if an individual contributed to a given pangenome" needs to be discussed properly. The discussion says: "PanMixer obfuscates haplotype paths for selected individuals, enabling participation in the pangenome reference without the loss of privacy.", which is only true for the stricter, former question. The authors could and should do a much better job in discussing the limitations of the privacy guarantees their work sets forth.
Author Response	We thank the reviewer for raising an important distinction. However, we believe that this concern stems from a misunderstanding of our methodology and we apologize for not being clearer. First, we would like to highlight that, mathematically, linkage attacks work symmetrically. Having said that, we have now conducted an additional attack to demonstrate that PanMixer prevents inference of the target individual's participation in the pangenome graph.<br><br>In this attack scenario, an attacker has access to the original genome of a target individual and wants to learn if the target individual is inside the pangenome graph. We empirically model this by linking the

	original genome of the target individual to the obfuscated pangenome graph.<br><br>Our results showed that at the same privacy threshold ($\varepsilon_{private} = 0.002$), this attack also failed. Specifically, many genomes within the pangenome achieved a higher linking score than the obfuscated genome. Furthermore, the linking score between the original and obfuscated genome falls near the center of the overall score distribution across all genomes, rather than appearing as an outlier (please see figure below). This indicates that the obfuscated individual is statistically indistinguishable from other genomes in the pangenome and is therefore effectively protected from this attack.

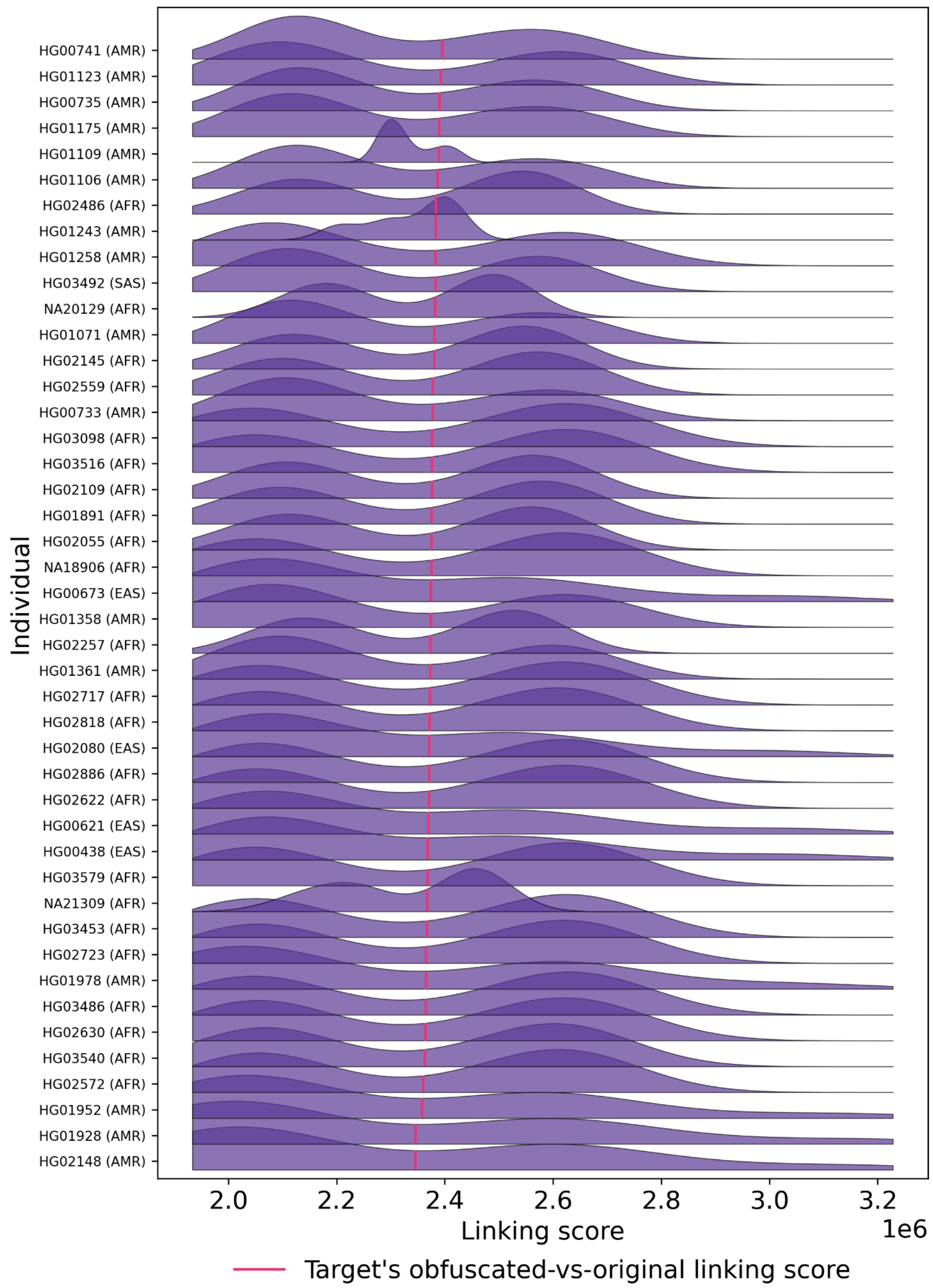

We would like to clarify that **our linking framework upweights rare alleles in both attacks**. Specifically, as described in the manuscript, the linking scores are constructed such that rarer alleles make larger contributions to the overall score, following prior linkage studies [15, 17, 16, 39]. This aligns with our obfuscation methodology which obfuscates rare variants first as those would have higher privacy risk.

	We now added the details of this additional attack and results to the manuscript. We also added additional clarification to the Discussion.
Excerpt From Revised Manuscript	**Section 2.4: Privacy metric aligns with linkage attack and genome reconstruction risk**<br>As shown in Fig. 3A, the attacker attempts to link each obfuscated haplotype path to genomes in the1000 Genomes Project Phase 3 callset [1], using a rarity-weighted matching score that gives greater weight to the shared rare variants, similar to approaches used previously [15, 17, 16, 39]. That is, the linkage score is defined as the negative sum of the log-frequencies of the shared genotypes, ensuring that shared rare variants contribute more to the linking scores (see Methods section 4.3.1 for more details).<br><br>…<br>Next, we evaluated a scenario analogous to a membership inference attack, in which an adversary possesses the full original genome of a target individual and seeks to determine whether that individual is represented in the pangenome. We modeled this attack by having the adversary attempt to link the target's known genome to all haplotype paths in the pangenome graph using the same rarity-weighted matching metric explained in the re-identification attack. A linkage attack is considered successful when the score distribution contains a clear outlier: one candidate genome scores significantly higher than all others, making the attacker more likely to identify that candidate as the obfuscated genome and conclude that the individual is present in the pangenome. Conversely, obfuscation is successful when the true obfuscated genome does not emerge as the top-scoring outlier. In this case, either another candidate receives a higher linking score or no candidate is clearly distinguishable, making the attacker more likely to misidentify the target or fail to identify them with confidence.<br><br>As shown in Supplementary Figure 2, the linking score between each target's original and obfuscated genome (red line) falls within the bulk of the distribution of linking scores against other subjects, indicating the obfuscated individual is indistinguishable from other candidates in the pangenome.<br><br>.

Supplementary Figure 2

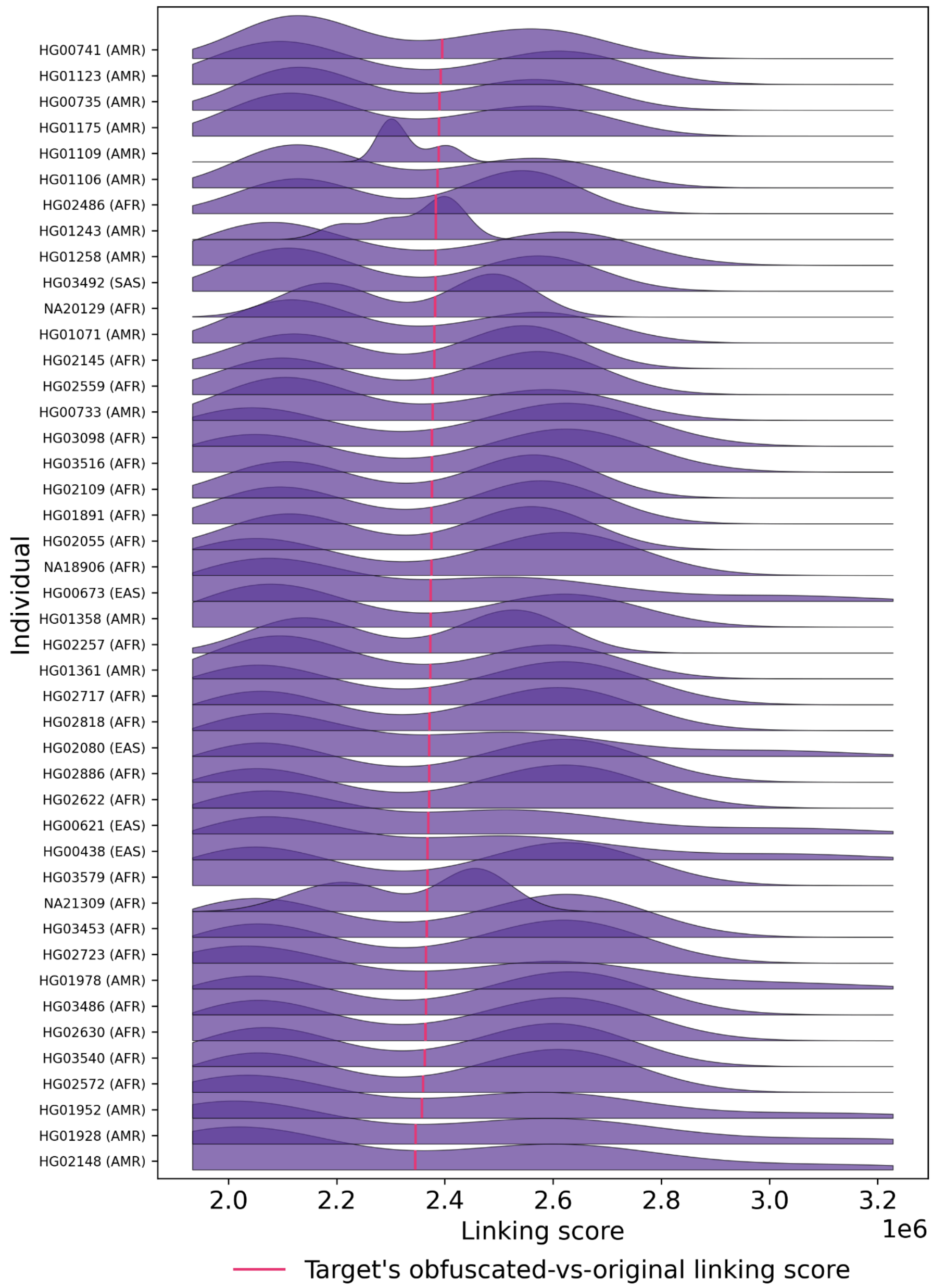

Supplementary Figure 2. Per-individual distribution of linking scores for an individual's original genome against all other subjects in a pangenome with this original genome obfuscated to $\varepsilon_{private} = 0.002$. For each row, the ridge summarizes the distribution of linking scores. The red line represents the linking score between the original and obfuscated genomes. A red line positioned within the bulk of the

	linking score distribution suggests limited ability for an attacker to discern whether the target individual was part of the pangenome.<br><br>**In Discussion:**<br><br>Crucially, the privacy guarantees provided by PanMixer are empirical and address realistic re-identification and membership inference attacks. It is possible that, with additional genomic information or knowledge of the parameters and obfuscation process itself, an attacker could perform stronger attacks than those PanMixer protects against. For large-scale, population-level references where identifiable haplotypes often discourage participation, PanMixer offers a practical solution for improving privacy by reducing both re-identification and membership inference risk. Providing formal, worst-case guarantees against membership inference remains a distinct and important direction for future work.

R1.2: Mathematical Definitions

Reviewer Comment	My second big issue with the paper is that the overview of the methods omits some crucial details in the main exposition. Specifically, the details of the haplotype probability calculation p(h) and the joint probability p(h, h') is not stated. I think it is particularly important to describe these functions in the main text because the paper largely rests on their details - their relationship defines precisely how we are to understand the privacy guarantees presented. I shouldn't have to go walking through the supplementary material to refer to a lemma to understand this. Similarly, I somewhat get the intuition about the obfuscation moves but I think this could be much clearer - and proper figure illustrating the moves would help. Overall, I think the writers should step back and try to explain the core ideas in the main text more precisely and with sufficient detail.
Author Response	We thank the reviewer for their comment, we have included definitions for all the probabilities as well as their purpose to help the reader understand the calculations without referencing the supplement. Please see excerpt below.

Excerpt From Revised Manuscript	**Section 2.2: Quantifying privacy and utility**<br>…We define the joint probability $p(h, h^*)$ blockwise over independent LD blocks $(b_1, \cdots, b_k)$. For blocks that are not obfuscated, the released and original haplotypes are identical, implying the joint probability \textit{of that block} equals the marginal probability ($p(h_{b_j}, h^*_{b_j}) = p(h_{b_j})$). Conversely, for obfuscated blocks, the released haplotype is sampled independently of the original, yielding a factorizable joint distribution ($p(h_{b_j}, h^*_{b_j}) = p(h_{b_j})p(h^*_{b_j})$). This construction leads directly to the blockwise decomposition of PMI derived in Supplementary Lemma 1.<br><br>**Section 2.2: Quantifying privacy and utility**<br>Because utility loss scales predictably with the number of edits, whereas privacy risk depends strongly on population allele frequencies, balancing the two requires a formal optimization framework. Each obfuscation move δ_j on an LD block b_j is associated with a utility cost $\eta_j = c(\delta_j)$ and a privacy risk $\epsilon_j = -\log p(h_{b_j})$ (derived in the Supplementary Lemma 1), where $p(h_{b_j})$ is the population probability of haplotype block h_{b_j}.

R1.3: Nit Picks 1

Reviewer Comment	"WPED is conceptually similar to the Weighted Graph Edit Distance (WGED), but instead of measuring edits between nodes and edges of two graphs, it measures edits between the original pangenome graph and the graph with obfuscated haplotype paths. A low WPED indicates few edits (high utility), whereas a high WPED indicates many edits (low utility)."<br><br>This is confusing, because in the prior paragraph you're talking about WPED in terms of individual haplotypes, not the whole pangenome.

Author Response	We thank the reviewer for this comment. We have revised the description of WPED to clarify that it measures the edits required to convert the paths within a pangenome graph to their obfuscated counterparts.
Excerpt From Revised Manuscript	**Section 2.2: Quantifying privacy and utility**<br>WPED is conceptually similar to the Weighted Graph Edit Distance (WGED), but instead of measuring edits between nodes and edges of two graphs,it measures edits between the paths in the original graph and the graph with the obfuscated genomes. A low WPED indicates few edits (high utility), whereas a high WPED indicates many edits (low utility).

R1.4: Nit Picks 2

Reviewer Comment	The read mapping section is good, although I think it is worth pointing out that mapping to pangenome graphs already either employs selective filtering or "personalized" subsetting of a given pangenome, i.e. it does not surprise me that the obfuscation moves, which seem likely to preserve structure, have only a very small effect on read mapping.
Author Response	We agree that we should point out that read mapping to pangenomes can be personalized by first producing a subsampling of the pangenome graph with individuals similar to the sequenced individual or removing any rare nodes in the graph and then performing mapping to this smaller graph. We have run additional benchmarks on pangenome graphs which were filtered and "personalized". Please see our updated Figure 5A.
Excerpt From Revised Manuscript	**Section 2.5.3: Obfuscation preserves read mapping quality**<br>… Notably, we evaluated several graph construction strategies commonly used to improve mapping performance, including full graphs, graphs filtered to remove variants with alternative allele frequency below 10%, and personalized graphs constructed using 8 haplotypes [1].<br><br>…

Fig. 5A reports the percentage of perfectly aligned reads (all bases matched), gaplessly aligned reads (matched without any indels), and reads with a mapping quality (MAPQ) of 60. Across all three metrics and all three constructions, the distributions of mapping rates from PanMixer graphs closely overlap those of the corresponding original graphs: on the full graph, perfectly aligned reads averaged 77.67\% (vs. 77.70\% for the baseline), gapless reads 95.52\% (vs. 95.55\%), and reads with a MAPQ of 60 77.36\% (vs. 77.29\%).

Figure 5A

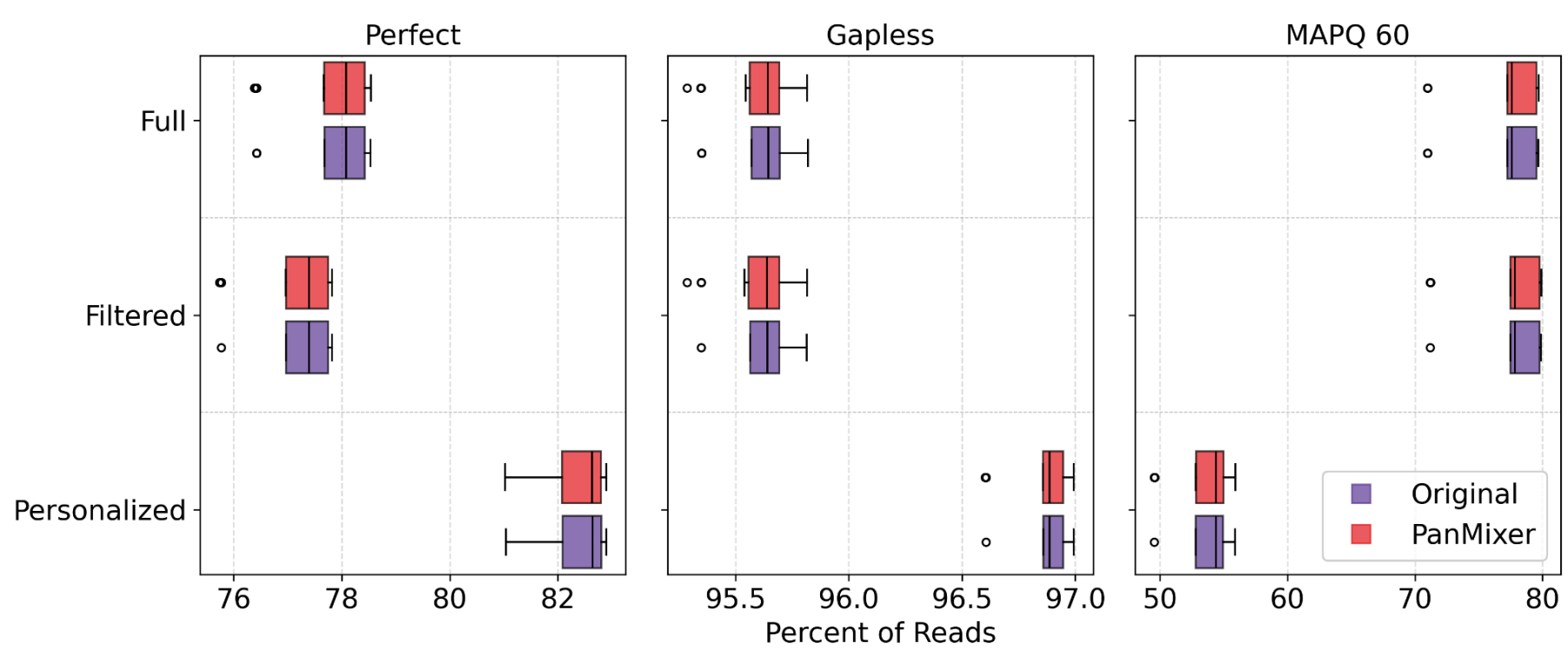

Figure 5: Downstream utility analysis for individuals outside of the pangenome reference. A: Percent of reads mapped exactly, gaplessly, or with MAPQ=60 on pangenome graphs generated by PanMixer and the original graph, evaluated across three graph constructions: the full pangenome, an allele-frequency-filtered pangenome (variants with alternative allele frequency < 10% removed), and a personalized pangenome [1]. We aligned 1000 Genomes individuals from five populations (EUR, EAS, AMR, SAS, AFR) against four PanMixer graphs, each obfuscating one target individual (HG00438, HG00733, HG02145, HG03492) at a maximum privacy risk of $\varepsilon_{private} = 0.002$, preventing linkage to the original. For each construction, PanMixer's mapping rates closely track those of the corresponding original graph, demonstrating that obfuscation preserves downstream alignment quality across all three pangenome regimes. Alignments were performed using vg giraffe (v1.68.0), and mapping statistics were obtained with vgtats…

	[1] Sirén, J., Eskandar, P., Ungaro, M.T. *et al.* Personalized pangenome references. *Nat Methods* 21, 2017–2023 (2024). https://doi.org/10.1038/s41592-024-02407-2

Reviewer 3's Strengths

> The authors present PanMixer, a framework that selectively obfuscates individual haplotype paths in a pangenome graph in order to reduce re identification risk while preserving the utility of the reference for downstream analyses (allele frequency estimation, linkage disequilibrium (LD) analysis, read mapping, etc.). The privacy utility trade off is cast as a 0-1 knapsack problem: each possible "obfuscation move" on an LD block carries a utility cost (Weighted Path Edit Distance, WPED) and a privacy benefit (point wise mutual information, PMI). The optimal subset of moves is obtained with an integer linear solver (OR Tools). Experiments on the draft human pangenome (47 individuals) demonstrate that (i) linkage based re identification attacks fail once the privacy metric ε falls below a threshold ($\varepsilon_{private} \approx 0.002$), (ii) genotype imputation attacks do not recover more information than a simple major allele baseline, and (iii) key downstream tasks show only negligible degradation (AF Wasserstein divergence < 0.01, LD L1 loss < 0.02, read mapping metrics within 0.1 % of the original). The authors argue that PanMixer will enable broader participation, especially from historically under represented populations, in future large scale pangenome projects.
>
> The manuscript tackles an important, timely problem with a solid methodological contribution and a thorough experimental validation. The concerns listed above are primarily about scalability, broader utility validation, and clarification of threat models. Addressing these points will significantly strengthen the paper.

We thank the reviewer for their feedback and are glad they believed the manuscript provides a logical and methodical contribution to pangenome privacy. We have addressed all the concerns below to strengthen the paper.

Reviewer 3's Comments

R3.1: Performance on Larger Pangenomes

Reviewer Comment	The scalability of the proposed method is not sufficiently addressed. In this study, experiments are conducted using

	haplotypes from only 47 individuals. Provide runtime/memory benchmarks for a realistic scenario (e.g., 350 genomes).
Author Response	We thank the reviewer for this suggestion. We have now included runtime and memory requirements for both v1 (44 individuals - HPRC released 47 genome assemblies but left 3 out for evaluation when producing the pangenome graphs) and the recent v2 (231 individuals) pangenome references from the HPRC.
Excerpt From Revised Manuscript	**Section 2.5.5: Performance of PanMixer**<br>Finally, we measured the computational efficiency and scalability of PanMixer by evaluating its performance on the HPRC v1 (n=44) and v2 (n=231) Minigraph-Cactus graphs (Table 1). Obfuscation took approximately 5 min and 14 GB memory on v1, and 78 min and 142 GB memory on v2. Note that the HMM haplotype probability estimation scales quadratically with the number of haplotypes in the graph.<br><br>(Table 1, below)<br><br>Table 1: Wall-clock time and peak memory to obfuscate a single subject in the HPRC v1 (44 total subjects) and HPRC v2 (231 total subjects) Minigraph-Cactus pangenome graphs. All 22 chromosomes are run in parallel; the reported time and memory are from the longest-running chromosome. Benchmarks were run on an HPC cluster with an AMD EPYC 9654 96-core processors and 735 GB RAM per node.

	**HPRC v1** (44)		**HPRC v2** (231)	
	Time	Memory	Time	Memory
Obfuscation (per subject)	5 min	14 GB	78 min	142 GB

R3.2: Estimated Allele Frequencies

Reviewer Comment	Privacy metric sensitivity to allele frequency estimation – PMI relies on population allele frequencies derived from 1000 Genomes, PanGenie, and the graph itself. The impact of miss estimated frequencies (e.g., for under represented groups) on ε is unclear.

Author Response	We thank the reviewer for raising this important point. To address this, we have updated PanMixer to more conservatively handle missing allele frequencies. Specifically, for alleles not found in 1000 Genomes or in the PanGenie callset, we now estimate the frequency using the pangenome graph itself: the numerator is the number of pangenome haplotypes carrying the allele, and the denominator is the number of 1000 Genomes haplotypes plus the number of pangenome haplotypes not represented in 1000 Genomes. This yields the allele frequency over the combined reference population, and ensures that variants observed in the pangenome but absent from 1000 Genomes are treated as rare, consistent with the fact that the pangenome contains substantially fewer individuals than 1000 Genomes<br><br>We have also updated the discussion to highlight the importance of accurate population-level allele frequency information for variants used in the pangenome graph. Please see the changes below.
Excerpt From Revised Manuscript	**Methods**<br>For novel variants not present in either dataset, frequencies are estimated conservatively: the numerator is the number of pangenome haplotypes carrying the allele, and the denominator is the number of 1000 Genomes haplotypes plus the number of pangenome haplotypes not represented in 1000 Genomes. This approach ensures that variants rare enough to be absent from standard callsets, but present in the pangenome graph, are assigned a frequency that reflects their rarity relative to the combined reference population.<br><br>**Discussion**<br>We acknowledge that PanMixer relies on accurate population allele frequency and LD estimates to quantify privacy risk. These inputs may currently be less precise for underrepresented populations or novel variants. However, as pangenome references expand in size and diversity, these estimates will naturally improve. This growth does not impose a computational bottleneck, as PanMixer scales linearly with the number of LD blocks and variants, independent of the total reference cohort size.

R3.3: Consent Implications

Reviewer Comment	The authors did not discuss how participants are informed about the obfuscation process or how consent forms should be adapted. Add a brief discussion on consent implications, e.g., whether participants need to be told that their haplotypes will be altered, and how this aligns with existing data sharing policies.
Author Response	We thank the reviewer for this thoughtful comment. We agree that consent and participant awareness are critical considerations in any privacy-preserving genomic framework.<br><br>In our setting, obfuscation is not applied by default; rather, it is performed only for participants who explicitly opt in to having their genomes obfuscated prior to data release. The goal of PanMixer is to expand participation by providing an additional privacy-preserving option for individuals who may otherwise be reluctant to contribute their data to open pangenome resources. As such, we envision that consent processes would clearly communicate both the risks of standard data release and the availability of obfuscation as a protective mechanism.<br><br>We agree that consent forms should be adapted to explicitly state that, if chosen, an individual's haplotypes may be algorithmically modified before release in a way that reduces re-identification risk while preserving population-level utility. This is aligned with existing data sharing practices that already incorporate controlled access, de-identification, or data transformation to mitigate privacy risks, but extends them to the level of haplotype representation in pangenome graphs.<br><br>We now add the following sentence to the Discussion:
Excerpt From Revised Manuscript	**Discussion**<br><br>In practice, obfuscation would be applied only with participant consent, and consent forms should explicitly communicate that haplotype paths may be algorithmically modified prior to release to reduce re-identification risk, aligning with existing privacy-preserving data sharing practices while offering participants greater control over how their genomic data are represented.
